# Endothelial YAP Signaling Promotes Blood‐Spinal Cord Barrier Repair in Mice After Spinal Cord Injury

**DOI:** 10.1002/advs.76891

**Published:** 2026-07-31

**Authors:** Jiawei Wang, Yaozhi He, Yanjiao Wang, Jingjing Zhang, Qishun Liang, Mengxian Jia, Yumin Wu, Zongjie Yuan, Ziwei Fan, Yuxuan Li, Huihui Zhang, Qinjiao Fu, Ying Wang, Zhihui Huang, Honglin Teng

**Affiliations:** ^1^ Department of Orthopedics (Spine Surgery) The First Affiliated Hospital of Wenzhou Medical University Wenzhou Zhejiang China; ^2^ School of Pharmacy Hangzhou Normal University Hangzhou Zhejiang China; ^3^ Cixi Biomedical Research Institute Wenzhou Medical University Ningbo Zhejiang China; ^4^ Clinical Research Center Affiliated Hangzhou First People’s Hospital Westlake University School of Medicine Hangzhou Zhejiang China; ^5^ The Fifth Affiliated Hospital of Wenzhou Medical University Lishui Zhejiang China

**Keywords:** ANGPT1, blood‐spinal cord barrier, Endothelial cells, PI3K/AKT, spinal cord injury, YAP

## Abstract

After spinal cord injury (SCI), the blood‐spinal cord barrier (BSCB) is disrupted, and endothelial cells, a critical component of BSCB, undergo proliferation and remodeling to maintain barrier integrity. However, the molecular mechanisms underlying BSCB remodeling remain unclear after SCI. Here, we reveal that Yes‐associated protein (YAP), a principal downstream effector of the Hippo signaling pathway, promotes endothelial proliferation and BSCB repair in mice after SCI. First, we found that YAP expression was significantly upregulated and activated in endothelial cells after SCI. Endothelial YAP knockout (YAP^TEK‐cre/ERT2^‐CKO mice) impaired endothelial proliferation, endothelial‐astrocyte end‐feet reorganization, and tight junction (TJ) integrity, thereby aggravating BSCB disruption and impairing BSCB remodeling and motor functional recovery after SCI. Mechanistically, endothelial YAP‐dependent BSCB repair was associated with angiopoietin‐1 (ANGPT1) expression and PI3K/AKT pathway activation after SCI. Exogenous ANGPT1 treatment mitigated YAP deficiency‐induced inhibition of endothelial proliferation and endothelial barrier disruption via PI3K/AKT signaling in vitro. Finally, activation of YAP signaling partially mitigated SCI‐induced BSCB disruption, ultimately improving motor function recovery. Together, these results identify endothelial YAP signaling as a critical regulator of BSCB remodeling associated with ANGPT1/PI3K/AKT pathway and provide a potential therapeutic target for SCI.

## Graphical abstract

Endothelial YAP is required for blood‐spinal cord barrier repair after spinal cord injury. YAP‐dependent vascular repair is associated with ANGPT1/PI3K/AKT signaling in vivo and may involve endothelial‐pericyte paracrine interactions. By supporting vascular remodeling, tight junction restoration, restored pericyte coverage, and astrocyte‐vascular reorganization, endothelial YAP contributes to improved neurological recovery, highlighting a potential therapeutic target for the injured spinal cord.

## 1 Introduction

Spinal cord injury (SCI) is a devastating neurological condition that frequently results in permanent motor, sensory, and autonomic dysfunction, imposing a substantial socioeconomic burden worldwide [1]. Despite advances in surgical decompression and rehabilitation strategies, effective treatments that promote significant neurological recovery remain limited [2]. The poor prognosis of SCI is largely attributed to complex secondary injury cascades that evolve after the initial mechanical insult, including vascular disruption, inflammation, and progressive neuronal degeneration [3, 4]. Therefore, identifying molecular mechanisms that regulate secondary injury and tissue repair remains a major challenge in the field.

Among the early pathological events following SCI, disruption of the blood‐spinal cord barrier (BSCB) is increasingly recognized as a central driver of secondary damage [5, 6]. The BSCB, composed of spinal microvascular endothelial cells forming tight junctions (TJs), basement membrane components, and perivascular glial cells, is essential for maintaining central nervous system homeostasis [7, 8, 9]. Structural disruption of endothelial TJs and increased vascular permeability permit the infiltration of inflammatory cells and circulating neurotoxic factors into the injured parenchyma, thereby amplifying neuroinflammation and neuronal loss [10]. Recent studies have begun to uncover the mechanisms governing BSCB disruption and restoration after SCI [5]. Acute barrier breakdown is largely attributed to inflammatory signaling, oxidative stress, and matrix metalloproteinase‐mediated degradation of TJ proteins and extracellular matrix components [11, 12]. In contrast, recovery of BSCB integrity during the subacute phase involves coordinated vascular remodeling processes, including endothelial survival, angiogenesis, pericyte stabilization, and astrocyte‐endothelial interactions that promote TJ reassembly [13]. Several signaling pathways, such as vascular endothelial growth factor (VEGF), Wnt/β‐catenin, and PI3K/AKT signaling, have been identified to be implicated in regulating endothelial barrier stability and vascular regeneration after SCI [14, 15, 16]. Importantly, accumulating evidence suggests that restoration of vascular integrity can mitigate secondary injury and improve functional recovery after SCI [10]. However, the key molecular regulators that orchestrate BSCB repair during the post‐injury phase remain unclear after SCI.

Yes‐associated protein (YAP), a principal downstream effector of the Hippo signaling pathway, functions as a transcriptional co‐activator that regulates cell proliferation, survival, mechanotransduction, and tissue regeneration [17]. In the vascular system, YAP activity has been shown to promote endothelial cell proliferation, angiogenic sprouting, and barrier stabilization in response to biomechanical and inflammatory stimuli [18, 19]. Endothelial YAP has also been implicated in protecting against vascular leakage and promoting tissue repair in pathological contexts such as ischemia and inflammation [20]. These findings suggest that YAP may function as a stress‐responsive regulator of vascular remodeling. Nevertheless, whether endothelial YAP participates in BSCB repair following SCI, and how it influences neurovascular remodeling in the injured spinal cord, remain unknown.

Angiopoietin‐1 (ANGPT1) and its receptor Tie2 are well‐established mediators of vascular stabilization and endothelial survival [21, 22, 23]. Activation of the downstream PI3K/AKT pathway enhances TJ assembly and promotes endothelial resistance to inflammatory injury [24, 25, 26]. In central nervous system disorders, activation of the ANGPT1/PI3K/AKT axis has been shown to protect blood‐brain barrier (BBB) integrity and attenuate vascular leakage [27]. Recent studies suggest that YAP plays an important role in vascular homeostasis and endothelial barrier regulation by transcriptionally modulating angiogenic factors, including components of the ANGPT1/Tie2 pathway [28, 29]. However, whether this signaling cascade mediates endothelial YAP‐dependent BSCB repair after SCI remains unclear.

In the present study, we aim to examine the role of endothelial YAP signaling in BSCB remodeling following SCI and to elucidate the underlying molecular mechanisms. We found that injury‐induced activation of endothelial YAP was required for BSCB repair and associated with ANGPT1/PI3K/AKT signaling, thereby attenuating secondary injury and improving neurological recovery in mice after SCI. This study provides evidence that endothelial YAP acts as a central coordinator of neurovascular repair in association with ANGPT1/PI3K/AKT signaling in mice after SCI.

## 2 Results

### 2.1 Endothelial YAP was Upregulated and Activated and its Knockout Aggravated BSCB Disruption in Mice after SCI

**FIGURE 1:**
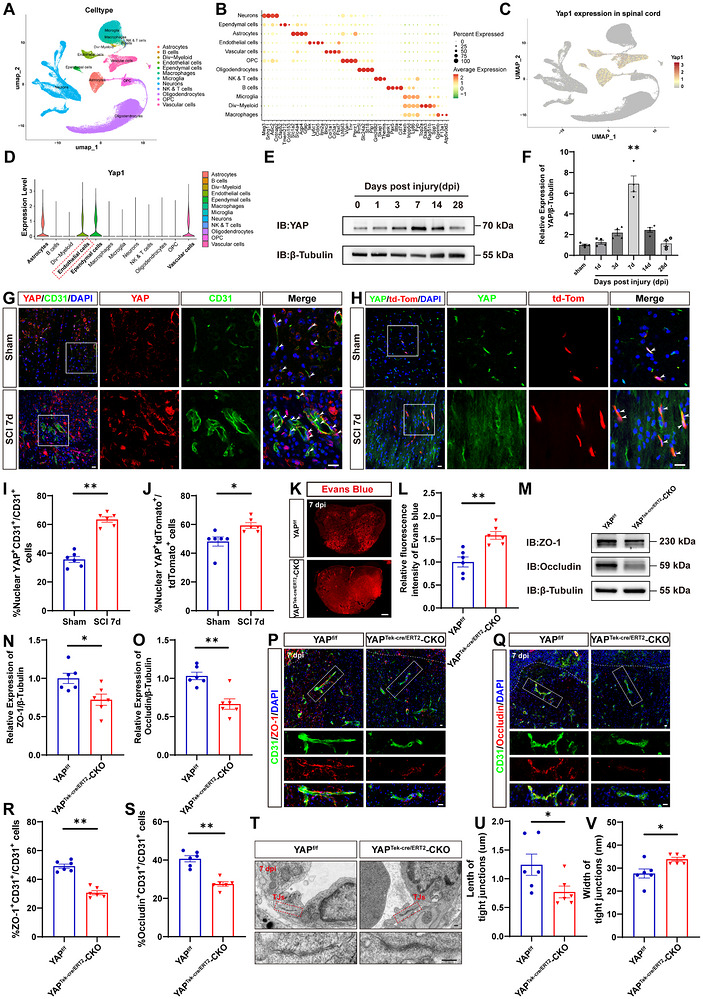
Endothelial YAP was upregulated and activated and its knockout aggravated BSCB disruption in mice after SCI. (A) Uniform Manifold Approximation and Projection (UMAP) plot showing clusters of spinal cord cells after spinal cord injury (SCI) obtained from the GEO database (GSE234774). (B) Dot plot showing marker genes used for cell type annotation in (A). (C) UMAP plot showing YAP1‐expressing cells across spinal cord cell populations. (D) Violin plots quantifying *YAP1* mRNA expression levels across different cell types. (E) WB analysis showing YAP protein expression at different time points following SCI. (F) Quantification of the relative YAP protein levels as shown in (E) (n = 4 blots from 4 mice per group). (G,H) Representative immunofluorescence staining images of YAP (red) and CD31 (green, a marker of endothelial cells) or YAP (green) in tdTom^+^ cells (an endothelial cell reporter mice, TEK‐tdTom) at 7 days after SCI. Scale bars = 20 µm. (I,J) Quantitative analysis of the percentage of nuclear YAP^+^ cells in CD31^+^ endothelial cells (I) and tdTom^+^ cells (J) as shown in (G) and (H), respectively (n = 6 sections from 6 mice per group). (K) Immunofluorescence images of Evans Blue (EB) extravasation in YAP^f/f^ and YAP^TEK‐cre/ERT2^‐CKO mice at 7 days after SCI. Scale bars = 200 µm. (L) Quantification of the relative fluorescence intensity of EB as shown in (K) (n = 6 sections from 6 mice per group). (M) WB analysis of the expression of ZO‐1 and Occludin in YAP^f/f^ and YAP^TEK‐cre/ERT2^‐CKO mice at 7 days after SCI. (N,O) Quantitative analysis of the relative expression of ZO‐1 (N) and Occludin (O) as shown in (M) (n = 6 blots from 3 mice per group). (P,Q) Representative immunofluorescence staining images for CD31 (green) and ZO‐1 (red) (P) or Occludin (red) (Q) expression in YAP^f/f^ and YAP^TEK‐cre/ERT2^‐CKO mice at 7 days after SCI. Scale bars = 20 µm. (R,S) Quantitative analysis of CD31^+^ZO‐1^+^ (R) and CD31^+^Occludin^+^ (S) cells as a percentage of CD31^+^ cells (n = 6 sections from 6 mice per group). (T) Representative transmission electron microscopy (TEM) images of tight junction (TJ) ultrastructure in YAP^f/f^ and YAP^TEK‐cre/ERT2^‐CKO mice at 7 days after SCI. Red squares and red arrows indicated the TJs. Scale bars = 200 nm. (U,V) Quantification of the length (U) and width (V) of TJs as shown in (T) (n = 6 sections from 6 mice per group). Data were presented as mean ± SEM, one‐way ANOVA with Tukey's post hoc test (F) and two‐tailed unpaired Student's *t*‐test (I, J, L, N, O, R, S, U, and V), ^*^ *p* < 0.05, ^**^ *p* < 0.01.

To investigate the role of YAP signaling in endothelial cells following SCI, we re‐analyzed a publicly available scRNA‐seq dataset from mouse SCI models, obtained from the *Tabulae Paralytica* atlas [30], which provides single‐cell and spatial transcriptomic profiles of spinal cord tissues across multiple time points post‐injury. Cell populations including astrocytes, neurons, oligodendrocytes, oligodendrocyte precursor cells (OPCs), microglia, macrophages, dividing myeloid cells (Div‐Myeloid), endothelial cells, vascular cells (primarily pericytes and vascular leptomeningeal cells, VLMCs), ependymal cells, B cells and NK/T cells were annotated based on established marker genes (Figure 1A,B), and YAP expression was quantified across distinct cell types. Interestingly, among all analyzed populations, endothelial cells exhibited one of the highest levels of YAP expression, comparable to astrocytes and ependymal cells (Figure 1C,D).

To further confirm the expression patterns of YAP in endothelial cells after SCI, a spinal cord crush injury model was established in mice [31], and tissues were collected at different stages after SCI. As expected, consistent with the scRNA‐seq analysis, western blotting showed that YAP protein expression was significantly upregulated after SCI and peaked at 7 days post‐injury (dpi) (Figure 1E,F). Immunofluorescence staining further showed marked upregulation of YAP in CD31^+^ endothelial cells, with prominent nuclear localization at 7 dpi (Figure 1G). Quantitative analysis further showed that the percentage of YAP^+^CD31^+^ cells among total CD31^+^ endothelial cells was significantly increased at 7 days after SCI (Figure 1I), supporting enhanced endothelial YAP activation after SCI. To exclude non‐specific staining of the CD31 antibody, endothelial cell reporter mice (TEK‐Cre/ERT2; Rosa26^tdTomato/tdTomato^, TEK‐tdTom) were used following SCI. As shown in Figure 1H,J, YAP was also significantly upregulated in tdTom^+^ endothelial cells at 7 days after SCI. Collectively, these results suggest that YAP is upregulated and activated in endothelial cells after SCI.

Given that endothelial cells are a critical component of the BSCB [9], these results suggest that endothelial YAP activation may play an important role in BSCB repair following SCI. To further assess the functional role of endothelial YAP signaling and avoid developmental confounding effects, tamoxifen‐inducible, endothelial cell‐specific YAP conditional knockout mice (YAP^TEK‐cre/ERT2^‐CKO) were generated (Figure S1). Under normal physiological conditions, no significant differences were observed between adult YAP^f/f^ and YAP^TEK‐cre/ERT2^‐CKO mice in footprint analysis or open‐field locomotor performance (Figure S2A–F). Moreover, Nissl staining and immunostaining for GFAP and NeuN revealed comparable neuronal and glial cell densities between the two groups (Figure S2G–L), indicating that in the adult mice, endothelial YAP signaling is dispensable for normal spinal cord homeostasis.

In contrast, at 7 days after SCI, YAP^TEK‐cre/ERT2^‐CKO mice exhibited significantly increased Evans Blue (EB) extravasation compared with control mice (Figure 1K,L), indicating exacerbated BSCB disruption. To further confirm the imaging‐based assessment of BSCB permeability, spectrophotometric quantification of EB extravasation using a formamide extraction assay was additionally performed. Consistent with the fluorescence imaging results, EB content was significantly increased in spinal cord tissue of YAP^TEK‐cre/ERT2^‐CKO mice compared with YAP^f/f^ controls at 7 days after SCI (Figure S3). Western blot analysis further showed significantly reduced expression of TJ proteins, including ZO‐1 and Occludin, in YAP‐deficient mice at 7 days after SCI (Figure 1M–O). Consistently, co‐immunostaining revealed a pronounced loss of ZO‐1 and Occludin specifically within CD31^+^ vascular structures in YAP^TEK‐cre/ERT2^‐CKO mice at 7 days after SCI (Figure 1P–S). Ultrastructural analysis by transmission electron microscopy (TEM) further demonstrated shortened TJ length and widened inter‐endothelial gaps in YAP^TEK‐cre/ERT2^‐CKO mice compared with YAP^f/f^ controls at 7 days after SCI (Figure 1T–V). Together, these results suggest that endothelial YAP signaling is required for maintaining TJ integrity and preventing BSCB disruption in mice after SCI.

### 2.2 Endothelial YAP Deficiency Impaired the Motor Functional Recovery and Exacerbated Neuronal Loss and Axonal Degeneration in Mice after SCI

**FIGURE 2:**
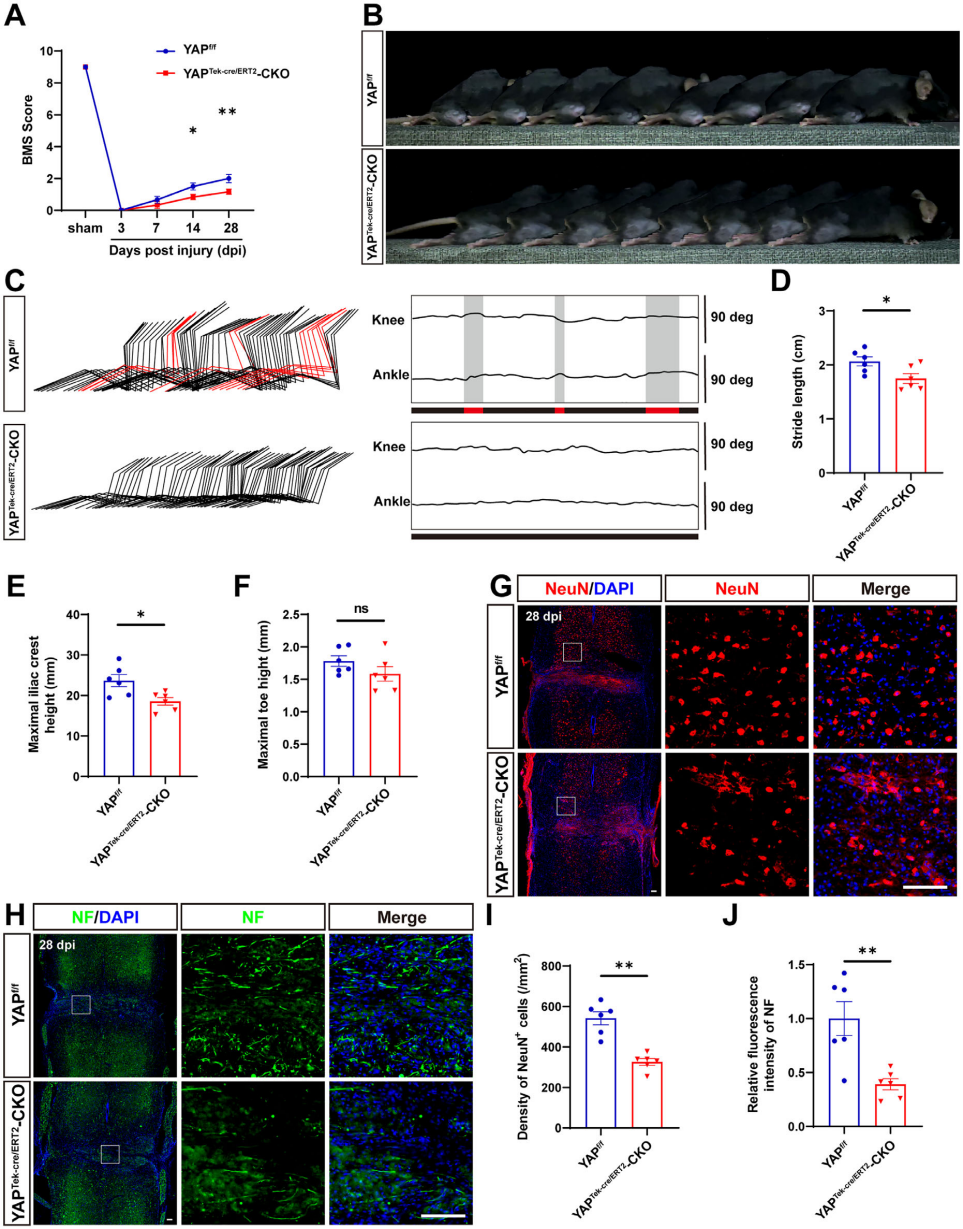
Endothelial YAP deficiency impaired the motor functional recovery and exacerbated neuronal loss and axonal degeneration in mice after SCI. (A) Analysis of BMS score in YAP^f/f^ and YAP^TEK‐cre/ERT2^‐CKO mice at different stages after SCI (n = 6 mice per group). (B,C) Representative chronophotographs of mice (B), accompanied by corresponding color‐coded stick‐figure (C) decompositions of hindlimb movements and oscillation traces of knee and ankle joint angles in YAP^f/f^ and YAP^TEK‐cre/ERT2^‐CKO mice at 28 days after SCI. (D‐F) Quantification of stride length (D), maximal iliac crest height (E), and maximal toe height (F) using video‐based kinematic analysis as shown in (B,C) (n = 6 mice per group). (G) Representative images of NeuN (red) immunostaining in YAP^f/f^ and YAP^TEK‐cre/ERT2^‐CKO mice at 28 days after SCI. (H) Representative images of NF (green) immunostaining in YAP^f/f^ and YAP^TEK‐cre/ERT2^‐CKO mice at 28 days after SCI. (I) Quantitative analysis of the density of NeuN^+^ cells adjacent to the lesion site as shown in (G) (n = 6 sections from 6 mice per group). (J) Quantitative analysis of the relative fluorescence intensity of NF as shown in (H) (n = 6 sections from 6 mice per group). Scale bar = 100 µm. Data were presented as mean ± SEM, two‐way ANOVA with Tukey's post hoc test (A) and two‐tailed unpaired Student's *t*‐test (D‐F, I, and J), ^*^ *p* < 0.05, ^**^ *p* < 0.01, ns indicated not significant.

To evaluate the functional consequences of endothelial YAP deletion after SCI, locomotor recovery was assessed at 28 days after SCI. Basso Mouse Scale (BMS) scoring revealed that the hindlimb functional recovery was significantly impaired in YAP^TEK‐cre/ERT2^‐CKO mice after SCI (Figure 2A). Consistently, kinematic gait analysis showed that a complete hindlimb dragging phenotype was observed in YAP‐deficient mice, whereas control mice exhibited partial recovery with occasional knee lifting movements (Figure 2B–F). These results suggest that motor functional recovery is impaired in YAP^TEK‐cre/ERT2^‐CKO mice after SCI.

Histological analysis using Nissl and Oil Red O staining showed significantly enlarged lesion areas in YAP^TEK‐cre/ERT2^‐CKO mice at 28 days after SCI (Figure S4A–F). Immunofluorescence staining further revealed reduced GFAP immunoreactivity adjacent to the lesion site, suggesting attenuated reactive astrocytes, accompanied by increased accumulation of F4/80^+^ macrophages and Iba1^+^ microglia/macrophages around the lesion site in YAP^TEK‐cre/ERT2^‐CKO mice (Figure S4G–J). These findings suggest that endothelial YAP deficiency may disrupt the vascular microenvironment after SCI, thereby indirectly affecting astrocyte activation and inflammatory responses. Moreover, NeuN and NF staining showed a marked reduction in neuronal survival and axonal fibers surrounding the lesion core in YAP^TEK‐cre/ERT2^‐CKO mice at 28 days after SCI (Figure 2G–J).

Collectively, these results suggest that endothelial YAP signaling plays a critical role in limiting lesion expansion and preserving neuronal and axonal integrity, and promoting motor functional recovery in mice after SCI.

### 2.3 Endothelial YAP Signaling was Required for Vascular Remodeling of BSCB Through Promoting Injury‐Induced Endothelial Proliferation in Mice after SCI

**FIGURE 3:**
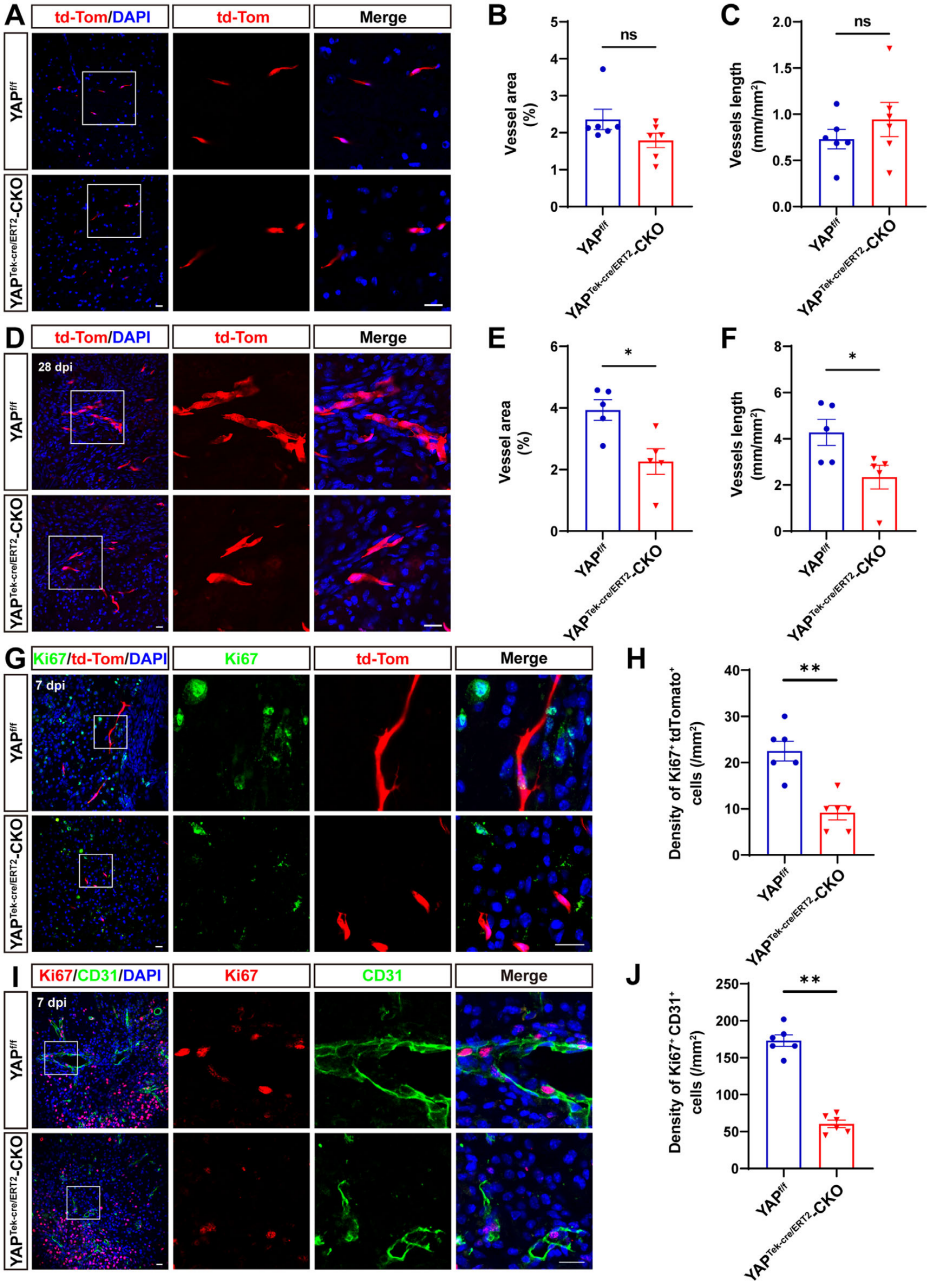
Endothelial YAP signaling was required for vascular remodeling of BSCB through promoting injury‐induced endothelial proliferation in mice after SCI. (A) Representative images of tdTom^+^ cells in control and YAP^TEK‐cre/ERT2^‐CKO‐tdTom mice. (B, C) Quantification of vascular area (B) and vascular length (C) of tdTom^+^ vessels as shown in (A), analyzed using the AngioTool software as previously described [72, 73] (n = 6 sections from 6 mice per group). (D) Representative images of tdTom^+^ cells in YAP^f/f^‐tdTom and YAP^TEK‐cre/ERT2^‐CKO‐tdTom mice at 28 days after SCI. (E,F) Quantification of Vessel area (E) and length (F) in tdTom^+^ cells as shown in (D) (n = 5 sections from 5 mice per group). (G) Representative immunofluorescence staining images of Ki67 (green) and tdTom^+^ cells in YAP^f/f^‐tdTom and YAP^TEK‐cre/ERT2^‐CKO‐tdTom mice at 7 days after SCI. (H) Quantitative analysis of the density of Ki67^+^ in tdTom^+^ cells as shown in (G) (n = 6 sections from 6 mice per group). (I) Representative immunofluorescence staining images of Ki67 (red) and CD31(green) in YAP^f/f^ and YAP^TEK‐cre/ERT2^‐CKO mice at 7 days after SCI. (J) Quantitative analysis of the density of Ki67^+^ in endothelial cells as shown in (I) (n = 6 sections from 6 mice per group). Scale bar = 20 µm. Data were presented as mean ± SEM, two‐tailed unpaired Student's *t*‐test, ^*^ *p* < 0.05, ^**^ *p* < 0.01, ns indicated not significant.

To further examine the role of YAP signaling in vascular remodeling of BSCB after SCI, TEK‐tdTom mice were used for endothelial lineage tracing and visualization (Figure S1). In the uninjured spinal cord, no significant differences were observed in the vascular area or vessel length of tdTom^+^ endothelial cells between adult control and YAP^TEK‐cre/ERT2^‐CKO mice (Figure 3A–C), indicating that endothelial YAP deletion did not affect endothelial homeostasis under basal conditions in adult mice. However, at 28 days after SCI, the percentage of tdTom^+^ endothelial cells was significantly reduced in YAP^TEK‐cre/ERT2^‐CKO mice compared with controls (Figure 3D–F), suggesting impaired vascular remodeling following injury in YAP^TEK‐cre/ERT2^‐CKO mice. Representative intermediary images were generated during AngioTool analysis, including vessel segmentation and skeletonization/tracing outputs used for vascular quantification (Figure S5). Given that YAP functions as a transcriptional co‐activator regulating cell proliferation and differentiation [17], we next examined endothelial proliferation after SCI. As expected, immunofluorescence staining at 7 days after SCI revealed a significant reduction in Ki67^+^ proliferating cells within both tdTom^+^ and CD31^+^ endothelial populations in endothelial YAP‐deficient mice (Figure 3G–J). Taken together, these results suggest that endothelial YAP signaling is a critical regulator of injury‐induced endothelial proliferation and vascular remodeling of BSCB in mice after SCI.

### 2.4 Endothelial YAP Deletion Inhibited Astrocyte‐Endothelial Reorganization and Reduced Astrocytic AQP4 Expression in BSCB after SCI

**FIGURE 4:**
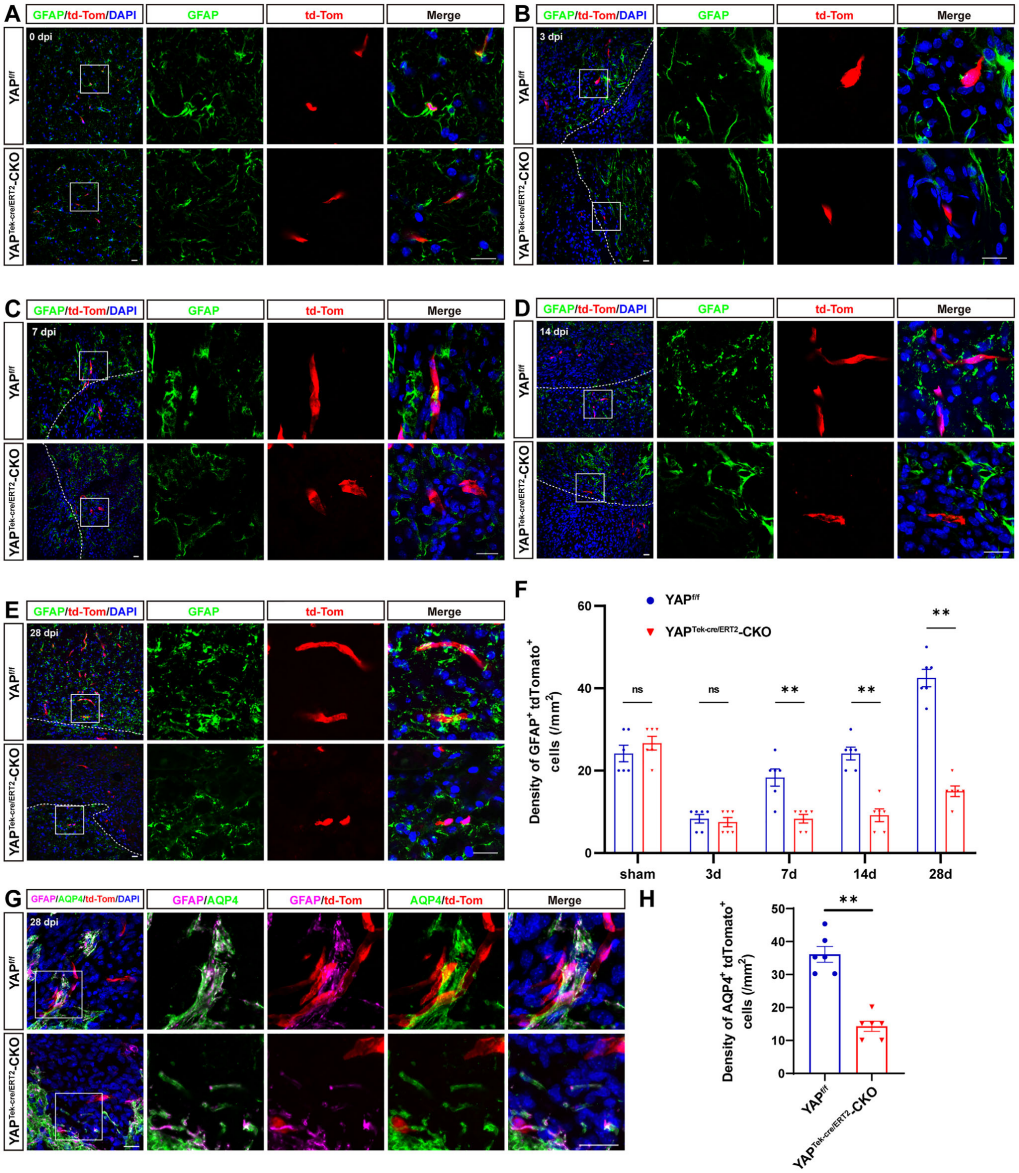
Endothelial YAP deletion inhibited astrocyte‐endothelial reorganization and reduced astrocytic AQP4 expression in BSCB after SCI. (A–E) Representative immunofluorescence staining images of GFAP (green) in control and YAP^TEK‐cre/ERT2^‐CKO‐tdTom mice at 0 d (A), 3 d (B), 7 d (C), 14 d (D), and 28 d (E) after SCI. (F) Quantitative analysis of the density of GFAP^+^ in tdTom^+^ cells at different time points following SCI as shown in (A‐E). Quantification was performed on the entire image field shown in (A‐E), whereas the enlarged panels represent selected regions for visualization only (n = 6 sections from 6 mice per group). (G) Double immunostaining of GFAP (far‐red) and AQP4 (green) in tdTom^+^ cells in YAP^f/f^‐tdTom and YAP^TEK‐cre/ERT2^‐CKO‐tdTom mice at 28 d after SCI. (H) Quantitative analysis of the density of AQP4^+^ in tdTom^+^ cells as shown in (G) (n = 6 sections from 6 mice per group). Scale bar = 20 µm. Data were presented as mean ± SEM, two‐tailed unpaired Student's *t*‐test, ^**^ *p* < 0.01, ns indicated not significant.

Astrocytes are essential components of the BBB, ensheathing endothelial cells through specialized end‐feet structures [32]. As the BSCB shares key cellular and molecular features with the BBB, we next examined whether endothelial YAP signaling was involved in astrocyte‐endothelial interactions to remodel BSCB after SCI. In sham‐operated mice, co‐immunostaining of GFAP and tdTom showed a tightly interwoven association between astrocytes and endothelial cells (Figure 4A). Following SCI, astrocytes initially detached from endothelial cells but gradually re‐established vascular contact and coverage during later stages of injury, consistent with the dynamic remodeling capacity of astrocytic end‐feet reported under conditions of vascular disruption [33, 34, 35]. Notably, this reorganization process was markedly impaired in YAP^TEK‐cre/ERT2^‐CKO mice compared with controls (Figure 4B–F). Astrocytic end‐feet expressing aquaporin‐4 (AQP4), a key regulator of BBB/BSCB function [36], were prominently detected during the recovery phase in control mice, but were significantly reduced in YAP‐deficient mice at 28 days after SCI (Figure 4G,H). These results indicate that endothelial YAP deletion impairs astrocyte‐endothelial interactions and reduces astrocytic AQP4 expression, thereby potentially compromising BSCB re‐establishment in mice after SCI.

### 2.5 Endothelial YAP Deficiency Suppressed ANGPT1 Expression and PI3K/AKT Signaling in Mice after SCI

**FIGURE 5:**
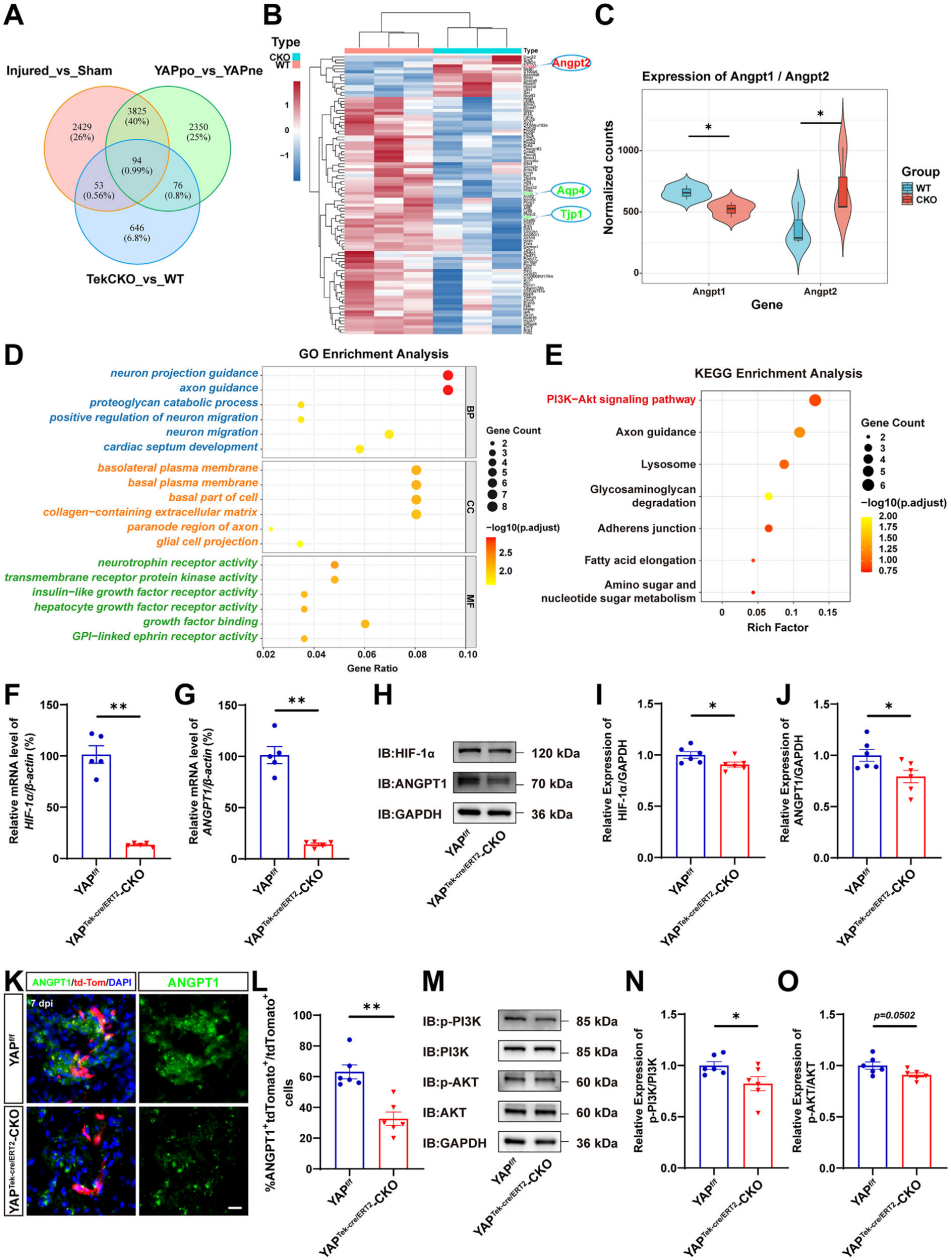
Endothelial YAP deficiency was associated with reduced ANGPT1 expression and PI3K/AKT signaling in mice after SCI. (A) Venn diagram of the overlapping differentially expressed genes between bulk RNA‐seq and scRNA‐seq datasets (GSE234774). (B) Heatmap of overlapping differentially expressed genes as shown in (A). (C) Differential expression of *angiopoietin‐1 (Angpt1)* and *angiopoietin‐2 (Angpt2)* in bulk RNA‐seq data. (D) The top 6 Gene Ontology (GO) terms enriched in the overlapping differentially expressed genes (from bulk RNA‐seq data). (E) The top 7 KEGG pathways enriched in the overlapping differentially expressed genes by gene ratio. (F,G) RT‐qPCR analysis of the relative mRNA levels of *HIF‐1α* (F) and *ANGPT1* (G) in YAP^f/f^ and YAP^TEK‐cre/ERT2^‐CKO mice at 7 days after SCI (n = 5 biological samples from 5 mice per group). (H) WB analysis of the expression of HIF‐1α and ANGPT1 in YAP^f/f^ and YAP^TEK‐cre/ERT2^‐CKO mice at 7 days after SCI. (I,J) Quantitative analysis of the relative expression of HIF‐1α (I) and ANGPT1 (J) as shown in (H) (n = 6 blots from 3 mice per group). (K) Representative immunofluorescence staining images for ANGPT1 (green) expression in YAP^f/f^‐tdTom and YAP^TEK‐cre/ERT2^‐CKO‐tdTom mice at 7 days after SCI. (L) Quantitative analysis of ANGPT1^+^tdTom^+^ cells as a percentage of tdTom^+^ cells as shown in (K) (n = 6 sections from 6 mice per group). (M) WB analysis of the expression of p‐PI3K, PI3K, p‐AKT, and AKT in YAP^f/f^ and YAP^TEK‐cre/ERT2^‐CKO‐tdTom mice at 7 days after SCI. (N,O) Quantitative analysis of the relative expression of p‐PI3K/PI3K (N) and p‐AKT/AKT (O) as shown in (M) (n = 6 blots from 3 mice per group). Scale bar = 20 µm. Data were presented as mean ± SEM, two‐tailed unpaired Student's *t*‐test, ^*^ *p* < 0.05, ^**^ *p* < 0.01.

To elucidate the molecular mechanisms underlying endothelial YAP‐mediated effects after SCI, bulk RNA sequencing was performed at 7 days after SCI. Differential expression analysis identified 483 upregulated and 386 downregulated genes in YAP^TEK‐cre/ERT2^‐CKO mice compared with controls. We further analyzed the differentially expressed genes by overlapping the bulk RNA‐seq results from control and YAP^TEK‐cre/ERT2^‐CKO mice with the scRNA‐seq comparisons of injured versus sham and YAP‐positive versus YAP‐negative endothelial cells, identifying 94 YAP‐associated injury‐related genes shared across these datasets, including 14 upregulated and 80 downregulated genes (Figure 5A). Heatmap visualization of these 94 intersection genes further revealed a transcriptional signature consistent with BSCB impairment, characterized by marked downregulation of *Tjp1 (ZO‐1)* and *Aqp4* mRNA (Figure 5B), both of which are critical for endothelial barrier integrity. Conversely, several genes involved in angiogenic and inflammatory responses were upregulated, with *Angpt2*, a key regulator of vascular remodeling and inflammation [37], showing a particularly prominent increase (Figure 5B). Previous studies have shown that HIF‐1α/YAP signaling promotes ANGPT1 expression, and that activation of the ANGPT1/PI3K/AKT pathway plays a crucial role in maintaining vascular stability and barrier integrity [26, 29]. Given the importance of this signaling axis in vascular homeostasis, we next examined whether ANGPT1‐associated signaling was impaired in endothelial YAP‐deficient mice during BSCB remodeling after SCI. RNA‐seq analysis revealed that *Angpt1*, but not *Angpt2*, was significantly downregulated in YAP‐deficient mice (Figure 5C). This result was further validated by qPCR analysis at 7 days after SCI, which confirmed a significant reduction in *Angpt1* and *HIF‐1α* mRNA expression (Figure 5F,G). Furthermore, immunostaining demonstrated that ANGPT1 was predominantly decreased in tdTom^+^ endothelial cells of YAP^TEK‐cre/ERT2‐CKO^ mice at 7 days after SCI (Figure 5K,L), suggesting that ANGPT1‐associated signaling was reduced in the endothelial compartment after endothelial YAP deletion during BSCB remodeling.

Consistently, Gene Ontology (GO) analysis revealed enrichment of cellular component terms associated with basement membrane structures, which are critical for BSCB integrity and astrocytic end‐feet anchoring (Figure 5D). Kyoto Encyclopedia of Genes and Genomes (KEGG) pathway analysis further identified the PI3K/AKT signaling pathway as one of the significantly affected pathways potentially related to ANGPT1‐associated vascular signaling (Figure 5E). Collectively, these findings suggest that endothelial YAP may contribute to the maintenance of vascular stability after SCI in association with ANGPT1/PI3K/AKT signaling.

We next investigated whether endothelial YAP deletion was accompanied by ANGPT1 expression and PI3K/AKT pathway activation in mice after SCI. As expected, at 7 days after SCI, WB analysis showed significantly reduced protein levels of HIF‐1α and ANGPT1 in YAP^TEK‐cre/ERT2^‐CKO mice compared with controls (Figure 5H–J). In line with these findings, WB assays at 7 days after SCI also showed reduced phosphorylation of PI3K (p‐PI3K) and AKT (p‐AKT), as indicated by lower p‐PI3K/PI3K and p‐AKT/AKT ratios in spinal cord tissue from YAP‐deficient mice (Figure 5M–O). Furthermore, increased YAP activation, ANGPT1 expression, and PI3K/AKT activation were predominantly restricted to the lesion epicenter, with substantially lower activation detected in adjacent rostral and caudal segments (Figure S6). Previous studies have reported functional crosstalk between YAP and HIF‐1α in regulating angiogenic responses [29], suggesting a possible link between YAP activity, HIF‐1α‐related signaling, and ANGPT1 expression. Taken together, these results suggest that endothelial YAP deficiency is associated with reduced ANGPT1 expression and attenuated PI3K/AKT signaling in mice after SCI, which may be responsible for the phenotype of aggravated BSCB disruption in YAP^TEK‐cre/ERT2^‐CKO mice after SCI.

### 2.6 ANGPT1 Mitigated YAP Deficiency‐Induced Inhibition of Endothelial Proliferation and Disruption of the Endothelial Barrier via PI3K/AKT Signaling In Vitro

**FIGURE 6:**
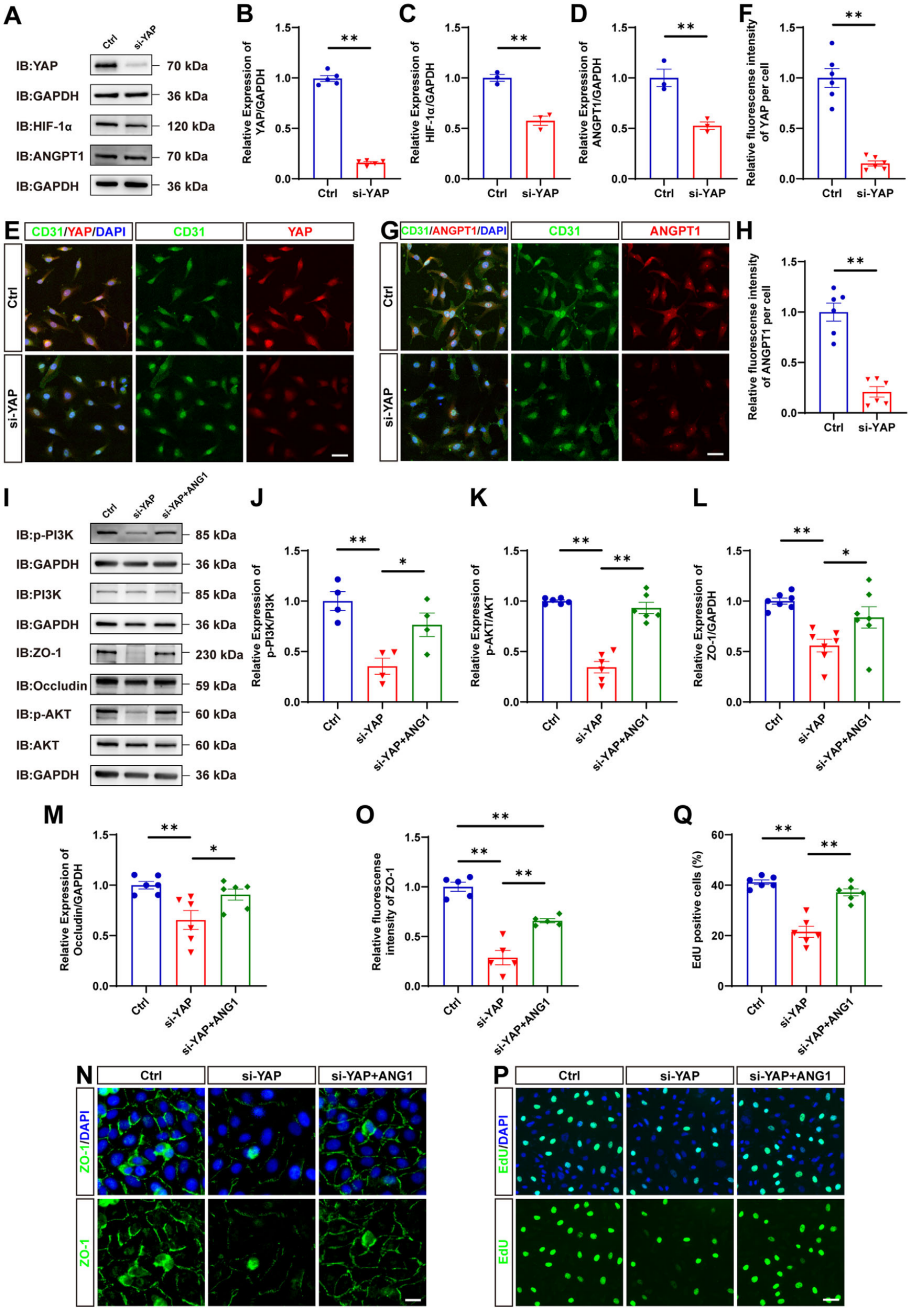
ANGPT1 mitigated YAP deficiency‐induced inhibition of endothelial proliferation and disruption of the endothelial barrier via PI3K/AKT signaling in vitro. (A) WB analysis of the expression of YAP, HIF‐1α, and ANGPT1 in control or YAP‐knockdown HUVECs after LPS. (B) Quantitative analysis of the relative expression of YAP as shown in (A) (n = 5 per group). (C,D) Quantitative analysis of the relative expression of HIF‐1α (C) and ANGPT1 (D) as shown in (A) (n = 3 per group). (E) Representative immunofluorescence staining images for YAP expression in control or YAP‐knockdown HUVECs after LPS. Scale bar = 50 µm. (F) Quantification of relative fluorescence intensity of YAP per cell as shown in (E) (n = 6 per group). (G) Representative immunofluorescence staining images for ANGPT1 expression in control or YAP‐knockdown HUVECs after LPS. Scale bar = 50 µm. (H) Quantification of relative fluorescence intensity of ANGPT1 per cell as shown in (G) (n = 6 per group). (I) WB analysis of the expression of ZO‐1, Occludin, p‐PI3K, PI3K, p‐AKT, and AKT in control, si‐YAP, and si‐YAP+ANG1 HUVECs after LPS. Cells in the rescue group were treated with recombinant ANGPT1 (500 ng/mL) for 48 h. (J–M) Quantitative analysis of the relative expression of ZO‐1, Occludin, p‐PI3K/PI3K (n = 4 per group), and p‐AKT/AKT as shown in (I) (n = 6 per group). (N) Representative immunofluorescence staining images for ZO‐1 expression in control, si‐YAP, and si‐YAP+ANG1 HUVECs after LPS. Scale bar = 20 µm. (O) Quantification of relative fluorescence intensity of ZO‐1 as shown in (N) (n = 6 per group). (P) Representative images of EdU‐stained HUVECs in control, si‐YAP, and si‐YAP+ANG1 HUVECs after LPS. Scale bar = 50 µm. (Q) Quantitative analysis of EdU^+^ cells as shown in (P) (n = 6 per group). Data were presented as mean ± SEM, two‐tailed unpaired Student's *t*‐test (B‐D, F, and H) and one‐way ANOVA with Tukey's post hoc test (J‐M, O, and Q), ^*^ *p* < 0.05, ^**^ *p* < 0.01.

To further examine the cell‐autonomous relationship between endothelial YAP and ANGPT1/PI3K/AKT signaling, we utilized small interfering RNA (siRNA) to knockdown YAP in human umbilical vein endothelial cells (HUVECs), and established the inflammatory cell model through lipopolysaccharide (LPS) stimulation for 24 h to mimic the inflammatory microenvironment in mice post‐SCI. The knockdown efficiency was subsequently confirmed by immunoblotting and immunostaining (Figure 6A–H). Notably, the expression of HIF‐1α and ANGPT1 was significantly reduced in si‐YAP‐HUVECs (Figure 6A–D), suggesting that YAP may regulate ANGPT1 expression in endothelial cells under in vitro conditions. To further determine whether ANGPT1 mediates the regulatory effects of YAP in endothelial cells, we performed rescue experiments by exogenous ANGPT1 treatment in si‐YAP‐HUVECs. Recombinant ANGPT1 was applied at a concentration of 500 ng/mL for 48 h based on previous studies [38]. As expected, WB analysis demonstrated that ANGPT1 treatment significantly increased the protein expression of ZO‐1 and Occludin in si‐YAP‐HUVECs, along with enhanced p‐PI3K and p‐AKT (Figure 6I–M). Consistent with the western blot findings, ZO‐1 immunofluorescence staining showed disrupted and fragmented junctional localization in si‐YAP‐HUVECs, whereas ANGPT1 treatment significantly restored junctional continuity and fluorescence intensity (Figure 6N,O). Furthermore, EdU incorporation assays further demonstrated that ANGPT1 treatment significantly increased the proliferation of si‐YAP‐HUVECs, as evidenced by a higher percentage of EdU^+^ cells compared with the si‐YAP group (Figure 6P,Q).

Collectively, these results indicate that ANGPT1 acts as a critical downstream effector of YAP to promote endothelial proliferation and prevent endothelial barrier disruption through activation of the PI3K/AKT signaling pathway in vitro.

### 2.7 Pharmacological Inhibition of MST1/2 to Activate YAP‐Associated Signaling Enhanced BSCB Repair and Functional Recovery in Mice after SCI

**FIGURE 7:**
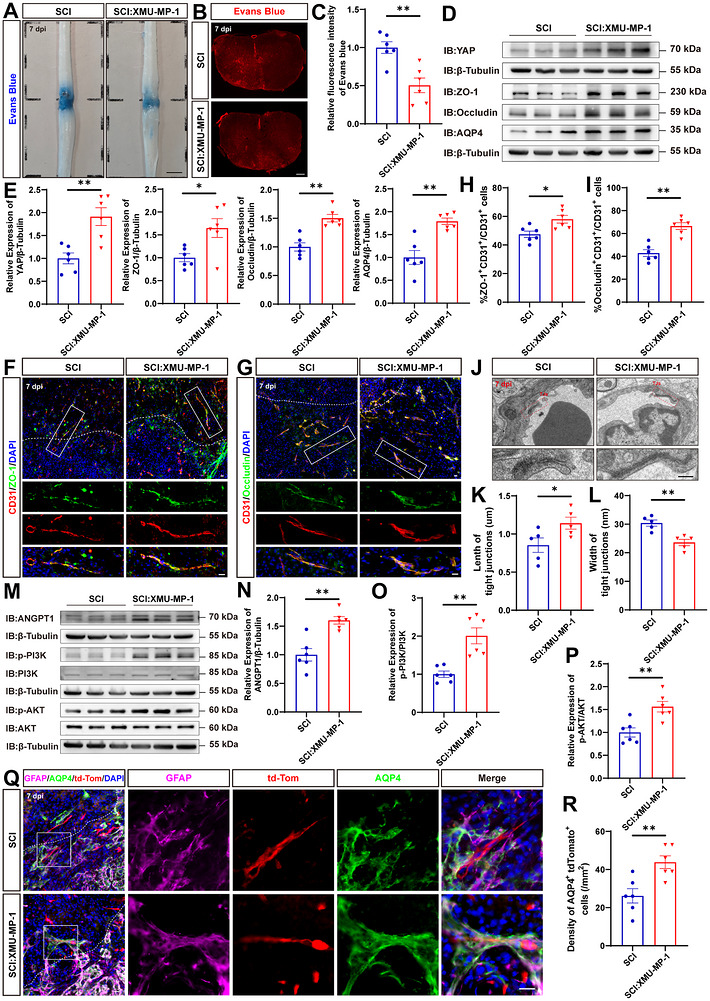
Pharmacological inhibition of MST1/2 to activate YAP‐associated signaling enhanced BSCB repair in mice after SCI. (A) Representative images of the spinal cord, showing EB extravasation in control and XMU‐MP‐1‐treated mice at 7 days after SCI. Scale bar = 2 mm. (B) Immunofluorescence images of EB extravasation in control and XMU‐MP‐1‐treated mice at 7 days after SCI. Scale bar = 200 µm. (C) Quantification of the relative fluorescence intensity of EB as shown in (B) (n = 6 sections from 6 mice per group). (D) WB analysis of the expression of YAP (GeneTex, GTX129151), ZO‐1, Occludin, and AQP4 in control and XMU‐MP‐1‐treated mice at 7 days after SCI. (E) Quantitative analysis of the relative expression of YAP, ZO‐1, Occludin, and AQP4 as shown in (D) (n = 6 blots from 3 mice per group). (F,G) Representative immunofluorescence staining images for CD31 (red) and ZO‐1 (green) (F) or Occludin (green) (G) expression in the spinal cord in control and XMU‐MP‐1‐treated mice at 7 days after SCI. Scale bar = 20 µm. (H,I) Quantitative analysis of CD31^+^ZO‐1^+^ (H) and CD31^+^Occludin^+^ (I) cells as a percentage of CD31^+^ cells as shown in (F) and (G), respectively (n = 6 sections from 6 mice per group). (J) Representative TEM images of TJ ultrastructure in the spinal cord in control and XMU‐MP‐1‐treated mice at 7 days after SCI. Red squares and red arrows indicated the TJs. Scale bars = 200 nm. (K,L) Quantification of the length (K) and width (L) of TJs as shown in (J) (n = 5 sections from 5 mice per group). (M) WB analysis of the expression of ANGPT1, p‐PI3K, PI3K, p‐AKT, and AKT in control and XMU‐MP‐1‐treated mice at 7 days after SCI. (N‐P) Quantitative analysis of the relative expression of ANGPT1 (N), p‐PI3K/PI3K (O), and p‐AKT/AKT (P) as shown in (M) (n = 6 blots from 3 mice per group). (Q) Double immunostaining of GFAP (far‐red) and AQP4 (green) in tdTom^+^ cells in control and XMU‐MP‐1‐treated mice at 7 days after SCI. Scale bar = 20 µm. (R) Quantitative analysis of the density of AQP4^+^ in tdTom^+^ cells as shown in (Q) (n = 6 sections from 6 mice per group). Data were presented as mean ± SEM, two‐tailed unpaired Student's *t*‐test, ^*^ *p* < 0.05, ^**^ *p* < 0.01.

To further assess the therapeutic relevance of YAP signaling through enhancing BSCB repair after SCI, mice were treated with XMU‐MP‐1, a selective MST1/2 inhibitor that has been reported to activate YAP signaling [39], and BSCB‐associated parameters were evaluated following XMU‐MP‐1 treatment (Figure 7). Interestingly, XMU‐MP‐1 significantly reduced EB extravasation at 7 days after SCI, indicating decreased BSCB permeability (Figure 7A–C). Consistently, spectrophotometric quantification of EB extravasation at 7 days after SCI using a formamide extraction assay demonstrated reduced EB content in spinal cord tissue following XMU‐MP‐1 treatment compared with YAP^f/f^ mice (Figure S3), further supporting improved BSCB integrity at the whole‐tissue level. Western blotting and immunofluorescence analyses at 7 days after SCI showed that XMU‐MP‐1 treatment significantly restored the expression of TJ proteins (Figure 7D–I). TEM analysis at 7 days after SCI further confirmed preservation of TJ ultrastructure following XMU‐MP‐1 treatment (Figure 7J–L). In addition, at 7 days after SCI, XMU‐MP‐1 treatment enhanced ANGPT1 expression and activated PI3K/AKT signaling, as demonstrated by western blot analysis (Figure 7M–P). Moreover, at 7 days after SCI, double immunostaining of GFAP and AQP4 in tdTom^+^ endothelial cells revealed enhanced astrocytic end‐feet coverage and closer astrocyte‐endothelial cell association after YAP activation (Figure 7Q,R). Collectively, these results indicate that pharmacological activation of YAP‐associated signaling may facilitate BSCB repair after SCI, potentially involving the ANGPT1/PI3K/AKT signaling axis.

From a broader perspective, XMU‐MP‐1 treatment significantly improved hindlimb locomotor recovery, as indicated by higher BMS scores compared with vehicle‐treated SCI mice (Figure S7A). Gait kinematic analysis further revealed increased stride length, maximal iliac crest height, and maximal toe height following XMU‐MP‐1 treatment (Figure S7B–F). Consistently, NeuN immunostaining demonstrated reduced neuronal loss in XMU‐MP‐1‐treated mice (Figure S7G,H). Double immunostaining of 5‐hydroxytryptamine (5‐HT) and GFAP showed enhanced serotonergic fiber penetration across the injury site and into caudal spinal cord segments (Figure S7I,J). Taken together, these results suggest that XMU‐MP‐1 treatment promotes BSCB repair and functional recovery in mice after SCI, with effects that are consistent with activation of YAP‐associated signaling.

## 3 Discussion

**FIGURE 8:**
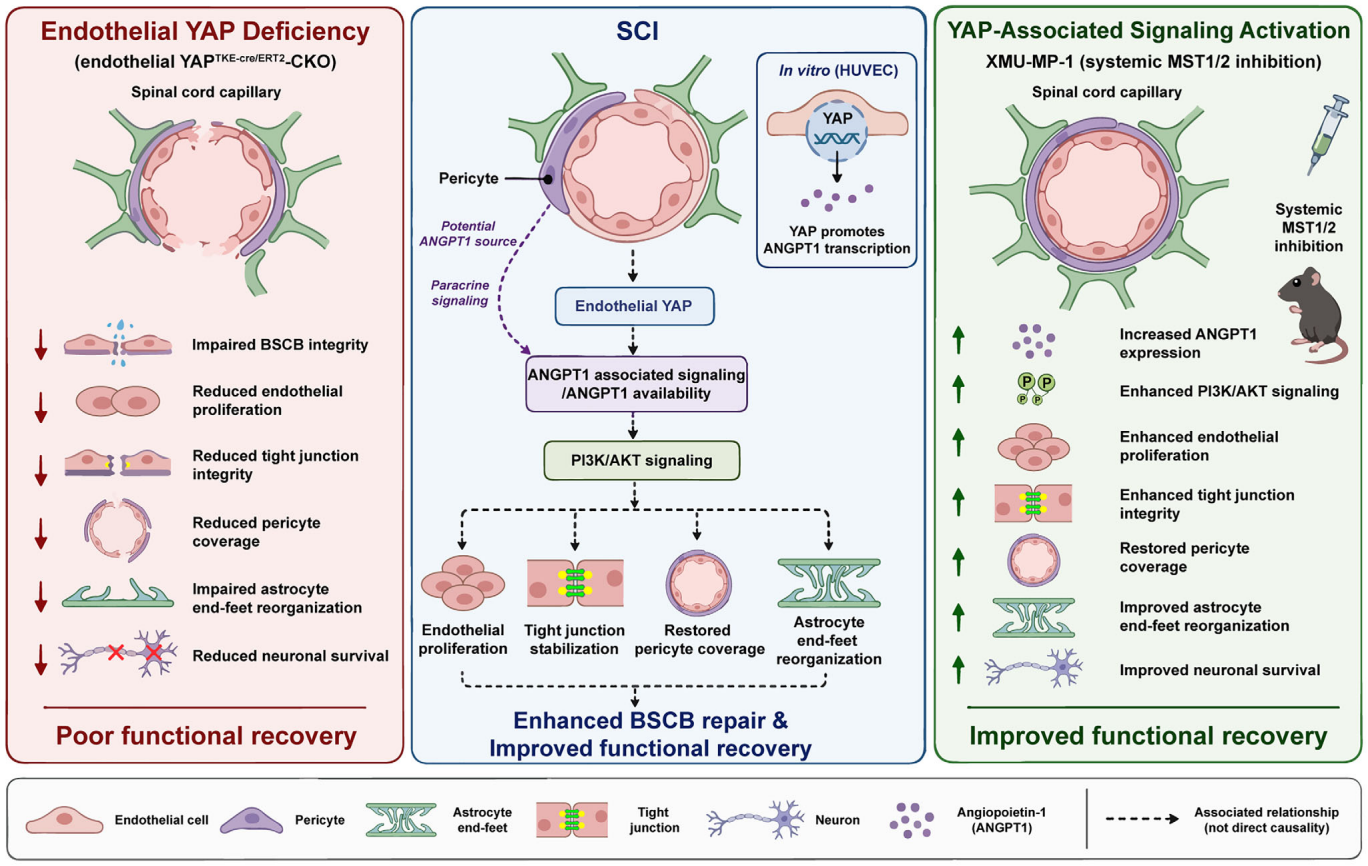
A working model of endothelial YAP‐dependent BSCB repair in mice after SCI in association with ANGPT1/PI3K/AKT signaling. Following SCI, endothelial YAP activation is required for BSCB repair and is associated with ANGPT1‐associated availability and PI3K/AKT signaling in vivo. Endothelial YAP deficiency (Left Panel): endothelial‐specific YAP deletion in YAP^TEK‐cre/ERT2^‐CKO mice results in impaired BSCB integrity, reduced endothelial proliferation, reduced tight junction integrity, reduced pericyte coverage, impaired astrocyte end‐feet reorganization, reduced neuronal survival, and poor functional recovery. YAP‐associated signaling activation (Right Panel): pharmacological inhibition of MST1/2 with XMU‐MP‐1 is associated with increased ANGPT1 expression, enhanced PI3K/AKT signaling, improved endothelial proliferation, enhanced tight junction stability, restored pericyte coverage, improved astrocyte end‐feet reorganization, improved neuronal survival, and improved functional recovery. In the in vivo model, pericytes are shown as a potential additional ANGPT1‐associated source, with a dashed paracrine arrow indicating a possible endothelial‐pericyte paracrine context. The cell‐autonomous relationship between YAP and ANGPT1 expression is shown separately in the in vitro HUVEC inset. These associated pathways may contribute to several cellular processes involved in barrier restoration, including endothelial proliferation, tight junction stabilization, restored pericyte coverage, and astrocyte end‐feet reorganization, thereby facilitating BSCB repair and functional recovery after SCI.

SCI triggers profound vascular disruption and BSCB breakdown, which contribute to secondary neuronal degeneration and functional impairment. In the present study, we identify endothelial YAP as a critical regulator of BSCB restoration and neurovascular remodeling following SCI (Figure 8). We demonstrate that YAP is markedly activated in endothelial cells following injury and that endothelial‐specific deletion of YAP impairs injury‐induced endothelial proliferation and astrocyte‐vascular reorganization, aggravates BSCB disruption, and ultimately exacerbates neuronal loss and locomotor deficits in mice after SCI. Mechanistically, our data indicate that endothelial YAP‐dependent BSCB repair is associated with ANGPT1 expression and PI3K/AKT signaling activation, which may contribute to vascular stabilization in vivo after SCI. Moreover, pharmacological activation of YAP by XMU‐MP‐1 significantly enhances BSCB repair and motor functional recovery after SCI. Collectively, our findings position endothelial YAP as a critical coordinator of post‐injury vascular regeneration and neuroprotection in SCI.

Increasing evidence suggests that Hippo‐YAP signaling plays a crucial role in regulating cellular responses to central nervous system (CNS) injury [31, 40, 41]. Our single‐cell transcriptomic analysis based on the publicly available dataset GSE234774 revealed that YAP was expressed across multiple spinal cord cell populations following injury, with notable enrichment in endothelial cells (Figure 1). In addition to endothelial and astrocytic populations, increased YAP expression was also observed in vascular‐associated cells, which mainly correspond to pericytes and, to a lesser extent, vascular leptomeningeal cells (VLMC) (Figure S8). Consistently, our additional PDGFRβ staining demonstrated that endothelial YAP deficiency reduced pericyte coverage, whereas YAP activation partially restored it (Figure S9). Given that pericytes are a major source of ANGPT1 and play essential roles in basement membrane assembly and vascular stabilization, these findings suggest that endothelial YAP may regulate BSCB repair not only cell‐autonomously but also by modulating pericyte recruitment and function. Furthermore, our co‐localization analysis indicates that ANGPT1 protein is associated with both endothelial and pericyte compartments, supporting a potential cooperative and paracrine mechanism within the neurovascular unit. However, the precise cell‐specific contribution of YAP in pericytes remains to be determined. These observations are consistent with previous reports showing that YAP signaling is dynamically activated in various neurological disorders and CNS injuries, including ischemic stroke and neurodegenerative diseases, where it regulates cellular stress responses and tissue repair processes [42, 43]. Studies from our group and others have demonstrated that YAP signaling contributes to neural stem cell activation, glial responses, and tissue remodeling in CNS injury contexts [31, 44, 45]. Extending these findings, our results highlight a previously underappreciated role of endothelial YAP signaling in coordinating vascular repair following SCI.

YAP, the major transcriptional co‐activator downstream of the Hippo pathway, has emerged as a key regulator of organ regeneration and tissue repair [46]. In the vascular system, YAP functions as a mechanosensitive transcriptional co‐activator that regulates endothelial proliferation, angiogenic sprouting, and vascular stability [47, 48]. Endothelial cells are highly responsive to mechanical and inflammatory cues generated during tissue injury, including shear stress alterations, extracellular matrix remodeling, and hypoxic stress [49, 50]. Consistent with these previous studies, in the present study, endothelial YAP deletion in adult mice did not significantly affect vascular organization under physiological conditions but markedly impaired endothelial proliferation following SCI (Figure 3). These findings suggest that YAP acts as a stress‐responsive regulator of regenerative angiogenesis rather than a determinant of steady‐state vascular maintenance. Such context‐dependent activation of YAP may allow endothelial cells to rapidly adapt to injury‐induced environmental changes and initiate vascular repair programs [51].

Restoration of the BSCB represents a crucial step in limiting secondary injury after SCI. BSCB disruption leads to infiltration of inflammatory cells, edema formation, and progressive neuronal damage [52]. TJ proteins such as ZO‐1 and Occludin are essential structural components of the barrier, and their loss contributes to vascular leakage during the early phase of injury [53]. In the present study, endothelial YAP deficiency resulted in increased EB extravasation, reduced expression of ZO‐1 and Occludin, and ultrastructural disruption of endothelial junctions, indicating that YAP signaling is required for maintenance of endothelial barrier integrity during the reparative phase (Figure 1). These findings are consistent with previous studies demonstrating that YAP contributes to vascular integrity and limits vascular leakage under pathological conditions [54]. Notably, the temporal scope of the present study is limited to the acute and subacute phases of SCI (7–28 dpi). Previous longitudinal studies have demonstrated that BSCB permeability follows a biphasic pattern after injury, characterized by an initial disruption, partial recovery within the first few weeks, and subsequent secondary increases in permeability in rostral and caudal regions, with more complete restoration occurring only at later time points (∼6–10 weeks post‐injury) [55, 56, 57]. In this context, our findings primarily reflect early barrier disruption and initial repair processes, rather than the full course of long‐term BSCB remodeling. Moreover, as YAP is a stress‐responsive signaling molecule, its activation dynamics may also change over time, and it remains unclear whether YAP signaling is sustained, diminished, or reactivated during later stages of injury and repair. Therefore, extended time‐course studies in the future will be required to fully define the role of YAP signaling in long‐term BSCB restoration and to determine whether the effects observed here persist during the later phases of vascular remodeling. In addition, although the present study primarily focused on changes in total YAP expression, assessment of YAP phosphorylation status and subcellular localization will provide further insight into the dynamic regulation and activation state of YAP signaling following SCI. Future studies incorporating these analyses will help to further clarify the precise mechanisms underlying endothelial YAP‐mediated vascular repair.

Beyond its direct effects on endothelial cells, our study reveals that endothelial YAP influences astrocyte‐vascular interactions, which are essential for barrier re‐establishment after SCI (Figure 4). Astrocytic end‐feet normally ensheath blood vessels and contribute to barrier stability through polarized expression of AQP4, a water channel involved in fluid transport and ionic homeostasis [36, 58]. We observed impaired astrocyte reattachment to blood vessels and reduced AQP4 expression in YAP‐deficient mice, suggesting impaired reconstruction of the astrocyte‐vascular interface. One possible explanation is that endothelial cells may secrete angiocrine factors and extracellular matrix components that guide astrocyte migration and end‐feet polarization. YAP‐dependent transcriptional programs may therefore regulate endothelial‐derived trophic signals that facilitate astrocytic attachment to the vascular wall. In addition, impaired endothelial repair itself may secondarily disrupt astrocyte polarization and AQP4 localization. Recent evidence suggests that pericytes play an important role in astrocyte‐endothelial communication and regulation of AQP4 polarization within the neurovascular unit. Notably, AQP4 expression is enriched in astrocytic end‐foot membranes adjacent to pericytes, supporting the concept that pericytes contribute to perivascular astrocytic organization and homeostasis [26, 59]. Consistent with these observations, our additional immunostaining demonstrated prominent co‐localization of ANGPT1 with PDGFRβ^+^ pericytes after SCI (Figure S9). Moreover, endothelial YAP deficiency reduced pericyte‐associated ANGPT1 expression, suggesting that endothelial YAP signaling may indirectly influence astrocyte‐vascular coupling and vascular remodeling by modulating the pericyte microenvironment and ANGPT1‐associated paracrine signaling. These findings indicate that endothelial YAP contributes not only to vascular regeneration but also to restoration of astrocyte‐vascular coupling during recovery after SCI.

The close interaction between endothelial cells and astrocytes underscores the importance of neurovascular unit (NVU) remodeling during spinal cord repair. The NVU is composed of endothelial cells, astrocytes, neurons, pericytes, and extracellular matrix components that collectively regulate barrier permeability and maintain CNS homeostasis [60]. Following SCI, disruption of NVU architecture, including endothelial junction breakdown, astrocytic end‐feet detachment, and loss of perivascular signaling contributes to barrier dysfunction and neuronal degeneration. Conversely, coordinated reconstruction of NVU components is increasingly recognized as a critical determinant of functional recovery. In the present study, we found that endothelial YAP deficiency was associated with reduced pericyte coverage of microvessels after SCI, whereas pharmacological activation of YAP signaling partially restored pericyte‐vessel association. These findings are consistent with previous studies demonstrating that pericyte‐derived ANGPT1 activates endothelial Tie2/PI3K/AKT signaling to promote vascular stabilization, barrier integrity, pericyte recruitment, and vascular maturation [21, 26, 61]. Together with our additional co‐localization analysis showing ANGPT1 expression in both PDGFRβ^+^ pericytes and tdTom^+^ endothelial cells after SCI, these results suggest that endothelial YAP may regulate BSCB repair not only through endothelial‐intrinsic mechanisms but also through modulation of endothelial‐pericyte paracrine signaling within the injured neurovascular niche. Therefore, in addition to regulating endothelial TJ integrity, endothelial YAP signaling may also facilitate reconstruction of the perivascular microenvironment by coordinating endothelial‐pericyte interactions and ANGPT1‐associated paracrine signaling, thereby promoting astrocytic reattachment, restoration of AQP4 polarity, and recovery of NVU structure and function after SCI (Figures 3 and 4, and Figure S9).

Mechanistically, our data identify the ANGPT1/PI3K/AKT pathway as an important downstream signaling associated with endothelial YAP‐dependent BSCB repair. ANGPT1 is a well‐established vascular stabilizing factor that promotes endothelial survival and TJ assembly through Tie2‐dependent activation of PI3K/AKT signaling [61, 62]. Activation of the ANGPT1/PI3K/AKT pathway has been shown to protect the BBB in multiple CNS disorders [27]. In our study, endothelial YAP deletion was associated with reduced ANGPT1 expression and attenuated PI3K/AKT phosphorylation after SCI, whereas ANGPT1 supplementation rescued junctional disruption and proliferative defects in YAP‐silenced endothelial cells (Figures 5 and 6). Moreover, recent studies suggest that YAP cooperates with hypoxia‐related signaling pathways, including HIF‐1α, to regulate angiogenic gene expression under stress conditions [29]. Consistent with this notion, we observed reduced HIF‐1α levels in YAP‐deficient mice, suggesting that YAP may cooperate with hypoxia‐responsive signaling pathways to regulate angiogenic gene expression. Together, these findings support a model in which YAP integrates hypoxic and mechanical cues to regulate vascular stabilization and barrier repair, in association with ANGPT1‐related signaling.

Importantly, pharmacological activation of Hippo pathway signaling using XMU‐MP‐1 was associated with improved vascular repair and neurological recovery after SCI (Figure 7, Figure S7). XMU‐MP‐1 treatment reduced BSCB leakage, enhanced TJ integrity, increased ANGPT1 expression, and was accompanied by elevated PI3K/AKT signaling, together with improved locomotor outcomes. These effects were further associated with reduced neuronal loss and increased serotonergic fiber penetration across the injury site. However, the interpretation of these pharmacological findings warrants caution. XMU‐MP‐1 is systemically administered and functions as an inhibitor of MST1/2 kinases, which regulate signaling pathways beyond YAP/TAZ, including FOXO signaling. Moreover, MST1 and MST2 have been shown to interact with and inhibit AKT1 in certain cellular contexts [63, 64]. Therefore, the observed activation of PI3K/AKT signaling and functional recovery could, at least in part, reflect YAP‐independent effects of MST1/2 inhibition. In addition, due to the systemic nature of XMU‐MP‐1 administration, its effects are unlikely to be restricted to endothelial cells. Previous studies have shown that YAP signaling regulates multiple cellular responses after SCI, including glial scar remodeling and activation of ependymal progenitor cells [31, 44, 45]. Therefore, the beneficial effects observed here may arise from coordinated responses across multiple cell types within the injured spinal cord microenvironment. Consistent with this, our genetic data identify endothelial YAP as an important contributor to vascular repair. However, the pharmacological effects of XMU‐MP‐1 should be interpreted as being supportive of a role for YAP signaling rather than definitive evidence of endothelial‐specific YAP activation. Future studies such as assessing the efficacy of XMU‐MP‐1 in endothelial‐specific YAP‐deficient models will be required to establish the extent to which its therapeutic effects depend on endothelial YAP signaling.

Several limitations of this study should be acknowledged. First, the causal role and cellular source of ANGPT1 downstream of endothelial YAP were not directly established in vivo. Because ANGPT1 is a secreted Tie2 ligand, protein co‐localization with endothelial or pericyte markers indicates compartmental association rather than definitive cellular production. Although our in vitro experiments in HUVECs support a cell‐autonomous relationship between YAP and ANGPT1 expression in endothelial cells, the in vivo decrease of ANGPT1 after endothelial YAP deletion may also reflect indirect endothelial‐pericyte crosstalk and altered perivascular ANGPT1 availability. Future studies using cell‐type‐specific *Angpt1* mRNA detection or conditional Angpt1 manipulation will be required to define the precise cellular source and causal contribution of ANGPT1 in endothelial YAP‐dependent BSCB repair. Second, although our results highlight the ANGPT1/PI3K/AKT pathway as an important downstream mechanism, YAP likely regulates additional transcriptional programs involved in extracellular matrix remodeling, inflammatory signaling, and metabolic adaptation after SCI. Future studies using genome‐wide chromatin profiling and single‐cell transcriptomics will further elucidate the endothelial YAP regulatory network. Third, although our endothelial‐specific knockout data demonstrate that endothelial YAP is required for BSCB repair after SCI, the present study does not fully define whether YAP activity in non‐endothelial cells, such as astrocytes, ependymal cells, or pericytes, contributes to injury responses or compensates for the loss of endothelial YAP. Additional cell‐type‐specific analyses will be needed to determine the contribution of non‐endothelial YAP signaling to post‐SCI vascular and neurovascular remodeling. Fourth, the long‐term consequences of sustained YAP activation beyond the subacute phase of SCI remain to be determined. Finally, the molecular mediators linking endothelial YAP activation to astrocytic AQP4 regulation require further investigation. In addition, due to the nature of the surgical procedures, blinding of the surgeon to genotype or treatment allocation was not feasible, which may have introduced potential bias.

## 4 Conclusions

Our study identifies endothelial YAP as a pivotal regulator of BSCB restoration and neurovascular remodeling following SCI. We demonstrate that injury‐induced activation of endothelial YAP promotes TJ stabilization, endothelial proliferation, and coordinated astrocyte‐vascular reorganization, thereby limiting secondary injury and preserving neuronal integrity. Mechanistically, these effects are associated, at least in part, with ANGPT1/PI3K/AKT signaling, linking Hippo pathway activity to vascular stabilization in the injured spinal cord.

Importantly, pharmacological activation of YAP enhances BSCB repair and improves functional recovery, underscoring the therapeutic potential of targeting endothelial YAP signaling in SCI. Together, our findings reveal a previously unrecognized role of endothelial YAP signaling in coordinating vascular and neural repair processes and provide a mechanistic framework for developing barrier‐focused therapeutic strategies in traumatic SCI.

## 5 Materials and Methods

### 5.1 Animal and Mice Breeding

To generate endothelial cell‐specific YAP knockout mice (YAP^TEK‐cre/ERT2^‐CKO), floxed YAP allele (YAP^f/f^) mice were crossed with TEK‐Cre/ERT2 transgenic mice (Shanghai Model Organisms). YAP^f/f^ mice were generated as previously described [65]. For lineage tracing and knockout validation, YAP^TEK‐cre/ERT2^‐CKO mice were crossed with Rosa26^tdTomato/+^ reporter mice (Shanghai Model Organisms) to obtain YAP ^f/f^; TEK‐Cre/ERT2; Rosa26^tdTomato/tdTomato^ mice (YAP^TEK‐cre/ERT2^‐CKO; tdTom). Parallel crosses between TEK‐Cre/ERT2 and Rosa26^tdTomato/+^ mice generated TEK‐Cre/ERT2; Rosa26^tdTomato/tdTomato^ (TEK‐tdTom) reporter controls. For conditional knockout induction, adult mice were intraperitoneally injected with 4‐Hydroxytamoxifen (4‐OHT) (MCE, 75 mg/kg body weight for 5 consecutive days). All animal procedures were approved by the Animal Ethical and Welfare Committee of Hangzhou Normal University (HSD20220917).

### 5.2 SCI Surgical Procedures and Treatment

Spinal cord crush injury was induced at the thoracic level (T8) as previously described [66]. Briefly, mice were anesthetized with isoflurane, and a T7‐T9 laminectomy was performed to fully expose the spinal cord. The spinal cord was then compressed for 2 s using 0.3‐mm forceps [67]. Following hemostasis, the muscle and skin layers were sequentially sutured with 5‐0 surgical threads. After surgery, mice were kept warm until recovery from anesthesia and received intramuscular penicillin to prevent infection. Bladders were manually expressed twice daily until spontaneous urination resumed. Mice in the YAP agonist group were administered XMU‐MP‐1 (1 mg/kg; MedChemExpress, Y‐100526) dissolved in DMSO via intraperitoneal injection every 2 days [39]. Animals were randomly assigned to experimental groups prior to surgery. Group allocation was concealed from investigators involved in downstream data acquisition and analysis.

### 5.3 Behavioral Analysis

To assess locomotor recovery following SCI, the behavioral evaluations were performed at predetermined time points (0, 3, 7, 14, and 28 days post‐injury) following standardized procedures [68]. Behavioral analyses were performed by investigators blinded to group allocation, with at least two independent observers involved in scoring where applicable. Distinct color markings, blue for forelimbs and red for hindlimbs, were applied to facilitate kinematic tracking as mice traversed a paper‐covered runway. Stride parameters, including stride length and width, were quantitatively analyzed [31]. BMS scoring was performed independently by two blinded observers, and the average score was used for analysis.

Hindlimb functional recovery was further characterized using the Basso Mouse Scale (BMS), a validated 9‐point index (0 = complete paralysis; 9 = normal locomotion) that assesses five neurological domains: joint movement, interlimb coordination, paw placement, trunk stability, and tail posture [69].

For detailed kinematic analysis, mice were allowed to walk along a narrow runway (80 cm long and 4 cm wide). Bilateral hindlimb movements during overground locomotion were recorded at 60 frames per second using an iPhone camera, with reflective markers positioned on the iliac crest, hip, knee, ankle, and toe tip. In cases of limited hindlimb mobility, forelimb movements were used to define the gait cycle. Marker trajectories were extracted from recorded videos using DeepLabCut. Video‐based analyses (including DeepLabCut tracking and parameter extraction) were conducted with group identity masked. Quantitative parameters derived per gait cycle included maximal iliac crest and toe heights, stride length, and joint angle range of motion. Selected kinematic parameters were further processed in MATLAB to generate chronophotographs and hindlimb stick diagrams for visualizing motion trajectories [67].

Spontaneous locomotor activity was assessed in a square open‐field arena (50 × 50 × 50 cm). Mice were individually placed in the arena and allowed to freely explore for 10 min without prior habituation under sound‐attenuated conditions. Locomotor parameters, including total distance traveled and distance traveled within the peripheral zone, were automatically quantified using an overhead video‐tracking system (EthoVision XT 12, Noldus). Automated tracking outputs were analyzed with group information concealed.

### 5.4 Cell Culture

Human umbilical vein endothelial cells (HUVECs) were obtained from Oujiang Laboratory, School of Pharmacy, Wenzhou Medical University. Cells were cultured in Dulbecco's Modified Eagle's Medium (DMEM; C11995500BT, Gibco) supplemented with 10% fetal bovine serum (FBS; S‐FBS‐MX‐015, SERANA) and 1% penicillin/streptomycin (A8180, Solarbio). The incubator was set to 5% CO_2_ at 37°C. HUVECs were passaged every 3‐4 days until 80‐90% confluence was reached.

### 5.5 Western Blot (WB)

Total proteins were extracted from cells or tissues using RIPA buffer and incubated at 4°C for 30 min, followed by centrifugation at 12, 000 × g for 20 min. For spinal cord samples, a 5‐mm segment centered on the lesion epicenter (epicenter ± 2.5 mm) was collected at the indicated time points, and the lesion epicenter together with the adjacent penumbra region was pooled for biochemical analysis. Protein concentration was quantified with a BCA protein assay kit (Thermo Fisher Scientific). Lysates were mixed with 5 × loading buffer, denatured at 100°C for 10 min, and separated on 10% sodium dodecyl sulphate‐polyacrylamide gel electrophoresis (SDS‐PAGE) gels. Proteins were then transferred to the polyvinylidene fluoride (PVDF) membranes (Merck Millipore). The membranes were then blocked with 5% skim milk and incubated at 4°C overnight with the indicated primary antibodies. Detailed information regarding all primary and secondary antibodies used in this study was provided in Table S1. After extensive washing with Tris‐buffered saline with 0.1% Tween‐20 (TBST), membranes were incubated with the corresponding HRP‐conjugated secondary antibodies for 1 h at room temperature. Protein bands were visualized using an enhanced chemiluminescence (ECL) detection system (Bio‐Rad, 1705061) and imaged with the GelView 6000Plus imaging platform (BLT). Densitometric quantification of band intensities was performed using ImageJ software (NIH), with group information concealed during analysis.

### 5.6 Immunostaining

After mice were perfused sequentially with phosphate‐buffered saline (PBS) and 4% paraformaldehyde (PFA), the spinal cords were post‐fixed in 4% PFA for 24 h. Tissues were then cryoprotected in 15% and subsequently 30% sucrose solutions until they fully sank. The T8‐T10 thoracic spinal cord segments were sectioned into 20‐µm‐thick transverse and horizontal slices using a freezing microtome (CryoStar NX50, Thermo Scientific). For quantitative analyses, sections were systematically sampled at defined intervals (e.g., every 200 µm) spanning the lesion epicenter (epicenter ± 2.5 mm). A predefined number of representative sections (1‐2 sections per animal depending on the assay) were used for analysis. Regions of interest (ROIs) were predefined based on anatomical landmarks, including peri‐lesional gray matter and, where applicable, dorsal and ventral horns. For immunofluorescence staining, sections were refixed for 30 min, subjected to antigen retrieval in sodium citrate buffer at 65°C for 30 min, and then permeabilized and blocked with 5% BSA (#V900933, Sigma–Aldrich) containing 0.3% Triton X‐100 (#T8200, Solarbio) at room temperature for 1 h. Subsequently, sections were incubated overnight at 4°C with the indicated primary antibodies. Detailed information regarding all primary and secondary antibodies used for immunofluorescence staining was summarized in Table S1. After thorough washing, sections were incubated with species‐appropriate fluorescent secondary antibodies together with the nuclear dye DAPI (1:1, 000, #4083, CST) for 1 h at room temperature. Fluorescent images were acquired using either a fluorescence microscope (VS200, Olympus) or a confocal microscope (AX R MP, Nikon).

All images were acquired using identical microscope settings (e.g., laser power, gain, and exposure time) across experimental groups. Quantification of fluorescence intensity and cell counts was performed using ImageJ (NIH). Identical threshold parameters were applied across all groups for fluorescence intensity analysis. All image acquisition and quantitative analyses were performed in a blinded manner by independent investigators.

For cell staining, the HUVECs were washed with PBS once and fixed with 4% PFA for 30 min. Then cells were permeabilized and blocked in PBS containing 5% BSA and 0.1% Triton X‐100 for 1 h at room temperature. The cells were subsequently incubated at 4°C overnight with primary antibody. After washing three times with PBS, cells were incubated with the appropriate secondary antibodies and DAPI for 1 h.

### 5.7 Nissl Staining

Spinal cord sections were rinsed three times with double deionized water (DDW), followed by sequential immersions in 95% ethanol for 1 min and 100% ethanol for 1 min for preliminary dehydration. The sections were then briefly rinsed with DDW and incubated in 0.1% cresyl violet solution (Sigma–Aldrich) for 5 min at room temperature. After staining, the samples were differentiated in 95% ethanol for 2 min and further dehydrated in 100% ethanol for 3 min. Subsequently, the sections were cleared in xylene for 10 min to enhance optical transparency and mounted with neutral resin under coverslips. The stained sections were examined under a microscope (VS200, Olympus).

### 5.8 Oil Red O Staining

Oil Red O staining was performed as described previously [70]. Briefly, spinal cord sections were air‐dried, fixed in formalin, and washed under running tap water for 5 min. The sections were then immersed in 60% isopropanol for 1 min before staining with freshly prepared Oil Red O working solution (Solarbio) for 10 min. Following staining, the sections were briefly rinsed in 60% isopropanol and counterstained with alum hematoxylin (Solarbio) for 3 min. After thorough rinsing with distilled water, the sections were mounted in aqueous glycerine jelly. All stained sections were observed under a microscope (VS200, Olympus) using identical imaging settings.

### 5.9 Quantitative Real‐Time PCR (qRT‐PCR)

Total RNA extraction was performed using the RNA‐Quick Purification Kit (ES‐RN001, ES Science), followed by cDNA synthesis with HiScript III RT SuperMix (R323‐01, Vazyme). Quantitative PCR analysis was conducted on a CFX96 Touch system (Bio‐Rad) with ChamQ Universal SYBR Master Mix (Q711‐02, Vazyme) to assess mRNA levels of *HIF‐1α* and *ANGPT1*. The results were normalized to *β‐actin*. Relative quantification was determined via the 2^‐ΔΔCt^ method using validated primers. The primers used included the following:

*HIF‐1α* (forward: 5′‐TCTCGGCGAAGCAAAGAGTC‐3′; reverse: 5′‐AGCCATCTAGGGCTTTCAGATAA‐3′);
*ANGPT1* (forward: 5′‐TGCATTCTTCGCTGCCATTCT‐3′; reverse: 5′‐ATTGCCCATGTTGAATCCGGT‐3′);
*β‐actin* (forward: 5′‐GGCACCACACCTTCTACAATG‐3′; reverse: 5′‐GGGGTGTTGAAGGTCTCAAAC‐3′).

### 5.10 RNA Sequencing and Analysis of Published scRNA‐Seq Data

Total RNA was isolated from spinal cord tissues of YAP^f/f^ and YAP^TEK‐cre/ERT2^‐CKO mice using TRIzol reagent (Invitrogen), following the manufacturer's protocol. RNA purity (A260/A280 > 1.8; A260/A230 > 2.0) was verified using a Nanodrop 2000 spectrophotometer (Thermo Fisher Scientific). Libraries were prepared by Novogene, involving fragmentation of poly(A)‐enriched mRNA, reverse transcription, and size selection (370‐420 bp). Paired‐end sequencing (150 bp) was performed on Illumina NovaSeq 6000. Clean reads were aligned to GRCm38 (HISAT2), and gene expression (FPKM) was quantified (featureCounts). Differential expression and GO enrichment (*P* < 0.05) were analyzed with DESeq2 and Xiantao tool, respectively.

For further analysis, a published scRNA‐seq dataset (GSE234774) from mice with SCI was downloaded and processed in RStudio following the methods described in the original study [30].

### 5.11 BSCB Permeability

The permeability of the BSCB was evaluated by Evans Blue (EB) extravasation [71]. Mice received an intraperitoneal injection of 0.5 mL 2% EB solution (Aladdin, China) 4 h prior to tissue collection at 7 days after SCI. Gross images of the spinal cord were captured to visualize overall dye distribution. Fixed spinal cords were sectioned at 20 µm, and EB fluorescence was examined using a fluorescence microscope (VS200, Olympus). Quantification of relative fluorescence intensity was performed with ImageJ. For quantification, sections were sampled at defined intervals (e.g., every 200 µm) spanning the lesion epicenter (epicenter ± 2.5 mm), with 1‐2 representative sections analyzed per animal depending on the assay. Regions of interest (ROIs) were defined as peri‐lesional areas. Relative fluorescence intensity was quantified using ImageJ with identical threshold parameters applied across all groups. All analyses were performed in a blinded manner.

For spectrophotometric quantification of EB extravasation, spinal cord tissue encompassing the lesion epicenter (± 2.5 mm) was collected following transcardial perfusion with saline to remove intravascular dye. Tissue samples were weighed and incubated in formamide (F6287, MACKLIN) at 60°C for 48 h, followed by centrifugation at 20, 000 × g for 30 min. The absorbance of the supernatant was measured at 620 nm using a microplate reader (Thermo Fisher Scientific), and EB concentration was quantified using a standard curve and normalized to tissue weight (µg/g tissue).

### 5.12 Transmission Electron Microscopy (TEM)

TEM was performed to evaluate BSCB ultrastructure after SCI. Briefly, spinal cord tissue blocks (∼1 mm^3^) were obtained from the lesion epicenter‐centered region (epicenter ± 2.5 mm) and fixed in 2.5% glutaraldehyde, followed by post‐fixation in 2% osmium tetroxide. Samples were dehydrated through a graded ethanol series and embedded in epoxy resin. Ultrathin sections were prepared using a Leica ultramicrotome, mounted on copper grids, and stained with uranyl acetate and lead citrate. Ultrastructural features of endothelial cells and TJs were examined using a transmission electron microscope (Hitachi HT7800).

### 5.13 ANGPT1 Treatment (In Vitro)

Recombinant ANGPT1 (Novoprotein, C04C) was reconstituted in sterile distilled water according to the manufacturer's instructions and diluted in culture medium to the desired concentration. Cells were treated with ANGPT1 at a final concentration of 500 ng/mL for 48 h. Control groups received an equivalent volume of vehicle.

### 5.14 siRNA‐Mediated YAP Knockdown and LPS Stimulation in HUVECs

HUVECs were transfected with YAP‐targeting siRNA (si‐YAP; sense: 5′‐CGACCAAUAGCUCAGAUCCUU‐3′, antisense: 5′‐AAGGAUCUGAGCUAUUGGUCG‐3′) or negative control siRNA (si‐NC) using Namipo transfection reagent (TranSheepBio, China) according to the manufacturer's instructions. Lyophilized siRNA was dissolved in RNase‐free DEPC water to 20 µM. Cells were seeded in 6‐well plates at 1.2–2 × 10^5^ cells per well and cultured for 16–24 h to reach ∼50% confluence. For transfection, 6 µL siRNA and 6 µL Namipo reagent were mixed and incubated for 5‐10 min at room temperature to form complexes, then added to cells. After 6 h, the medium was replaced with fresh complete medium, and the cells were cultured for 24–48 h. Knockdown efficiency was confirmed by western blot at 48–72 h post‐transfection. For inflammatory stimulation, transfected cells were treated with LPS (Sigma–Aldrich, USA) at 10 µg/mL for 24 h. All procedures were performed under RNase‐free conditions with multiple biological replicates to ensure reproducibility.

### 5.15 EdU Assays

The proliferation ability of HUVECs was assessed using the Beyo‐Click EdU Cell Proliferation Kit (Beyotime). The procedure was performed in accordance with the manufacturer's guidelines. Following EdU staining, the cells were observed under a fluorescence microscope (VS200, Olympus). The percentage of EdU^+^ cells was determined by calculating the number of EdU^+^ cells relative to the total cell number.

### 5.16 Quantification of Vascular Morphology

Vascular morphology was quantified using AngioTool as previously described [72, 73]. Briefly, fluorescence images of tdTom^+^ vessels were imported into AngioTool to quantify vascular parameters, including vessel area and vessel length. Sections were sampled at defined distances relative to the lesion epicenter (epicenter ± 2.5 mm), with 1‐2 representative sections analyzed per animal depending on the assay. ROIs were defined consistently across all samples. All images were analyzed using the same threshold parameters to ensure consistency. Analyses were performed in a blinded manner.

### 5.17 Statistical Analysis

Data were presented as mean ± SEM. Statistical analyses were performed using GraphPad Prism 8.0 with unpaired Student's *t*‐test for two‐group comparisons, or one‐/two‐way ANOVA followed by Tukey's or Bonferroni post hoc tests for multifactor analyses. Significance was accepted at *P <* 0.05. Relative expression levels were calculated by normalizing each individual value to the mean value of the corresponding control group, which was set to 1, unless otherwise specified. For western blot analysis, target protein intensities were first normalized to the corresponding loading control before relative normalization to the control group. Unless otherwise specified, n represented the number of biological replicates (individual mice). For histological quantification, 1‐2 anatomically matched representative sections/fields per mouse were imaged and analyzed depending on the assay. Biological replicate numbers for each experiment were indicated in the corresponding figure legends. All data acquisition and analyses were performed under blinded conditions unless otherwise specified.

## Author Contributions

Conceptualization: Ying Wang, Zhihui Huang, and Honglin Teng; methodology: Jiawei Wang, Yaozhi He, Yanjiao Wang, Jingjing Zhang, Qishun Liang, and Mengxian Jia; validation: Jiawei Wang, Yaozhi He, and Yanjiao Wang; formal analysis: Jiawei Wang, Yaozhi He, and Yanjiao Wang; resources: Ying Wang, Zhihui Huang and Honglin Teng; data curation and investigation: Jiawei Wang, Yaozhi He, Yanjiao Wang, Jingjing Zhang, Qishun Liang, Mengxian Jia, Yumin Wu, Zongjie Yuan, Ziwei Fan, Yuxuan Li, Huihui Zhang, and Qinjiao Fu; bioinformatic analysis: Jiawei Wang, Yaozhi He, and Qishun Liang; Writing – original draft preparation: Jiawei Wang and Yaozhi He; Writing – review & editing: Jiawei Wang, Yaozhi He, Ying Wang, Zhihui Huang, and Honglin Teng; visualization: Jiawei Wang and Yaozhi He; supervision: Ying Wang, Zhihui Huang, and Honglin Teng; funding acquisition: Ying Wang, Honglin Teng, and Zhihui Huang. All authors have read and agreed to the published version of the manuscript.

## Ethics Statement

All animal procedures were approved by the Animal Ethical and Welfare Committee of Hangzhou Normal University (Approval No. HSD20220917).

## Conflicts of Interest

The authors declare no conflict of interest.

## Supporting information




**Supporting File**: advs76891‐sup‐0001‐SuppMat.doc.

## Data Availability

The data that support the findings of this study are available from the corresponding author upon reasonable request.
