# Supplementary material for: Endothelial YAP Signaling Promotes Blood‐Spinal Cord Barrier Repair in Mice After Spinal Cord Injury
Source: Adv Sci (Weinh). 2026 Jul 31:e76891. Online ahead of print. doi: 10.1002/advs.76891 (PMC13427235; doi:10.1002/advs.76891)
Supplement: Supplementary file 1 — Supporting File: advs76891‐sup‐0001‐SuppMat.doc. [file ADVS-9999-e76891-s001.doc]

**Supplementary Figures and Table**

**
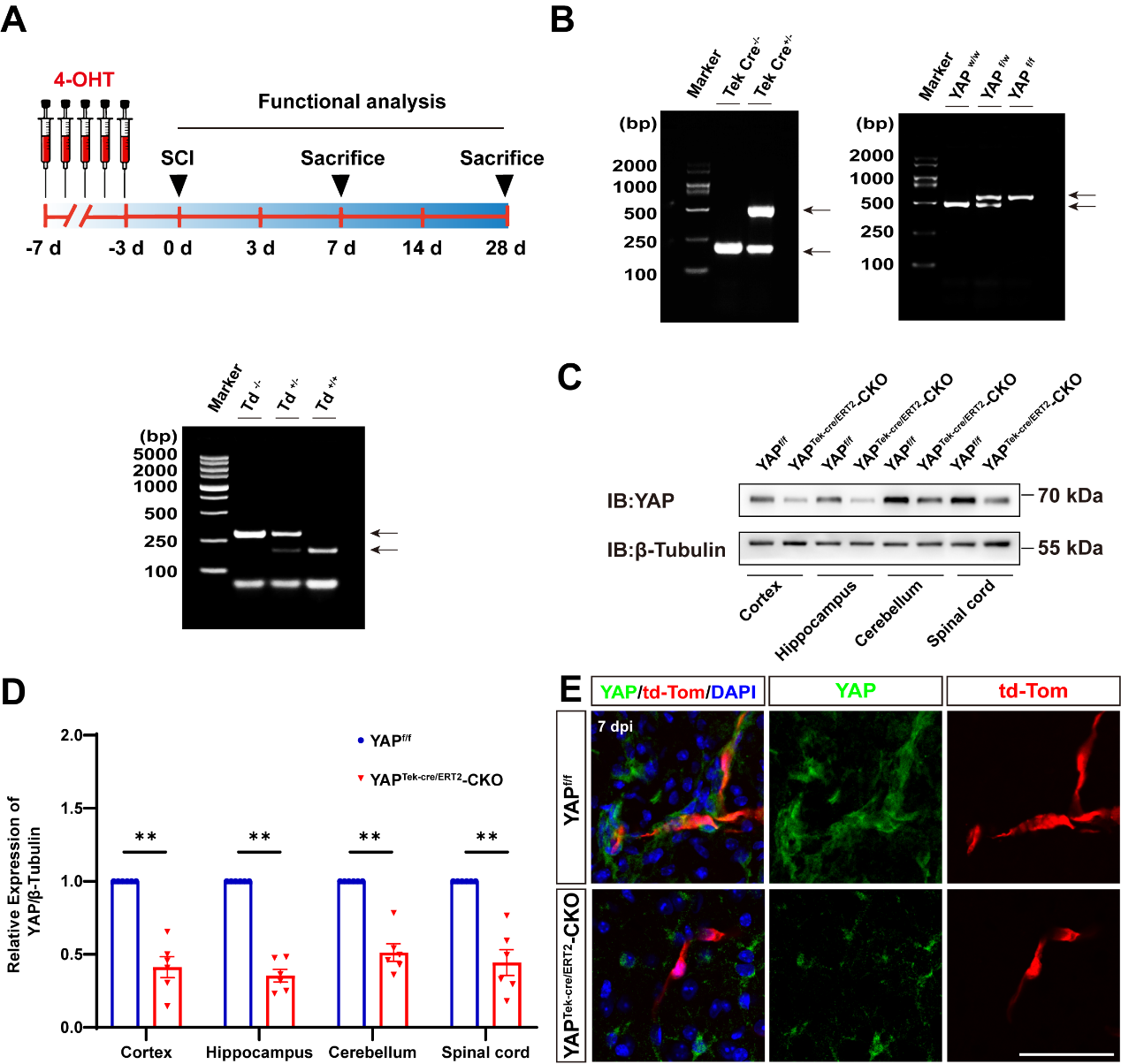
**

**Figure S1. Identification of YAP^TEK-cre/ERT2^-CKO-tdTom mice.**

**(A)** Experimental timeline illustrating 4-OHT administration, functional analyses, and behavior testing. **(B)** Genotyping results obtained via agarose gel electrophoresis. **(C)** Western blot detected YAP expression in the cortex, hippocampus, cerebellum, and spinal cord of 2-month-old YAP^f/f^ and YAP^TEK-cre/ERT2^-CKO mice. **(D)** Quantitative analysis of YAP expression as shown in (**C**) (n = 6 blots from 3 mice per group, normalized to YAP^f/f^ mice). **(E)** Double immunostaining analysis of YAP (green) and tdTom^+^ (red) in YAP^f/f^ and YAP^TEK-cre/ERT2^-CKO-tdTom mice. Scale bar = 20 μm. Data were presented as mean ± SEM, two-tailed unpaired Student’s t-test, ^**^ *p* < 0.01.

**
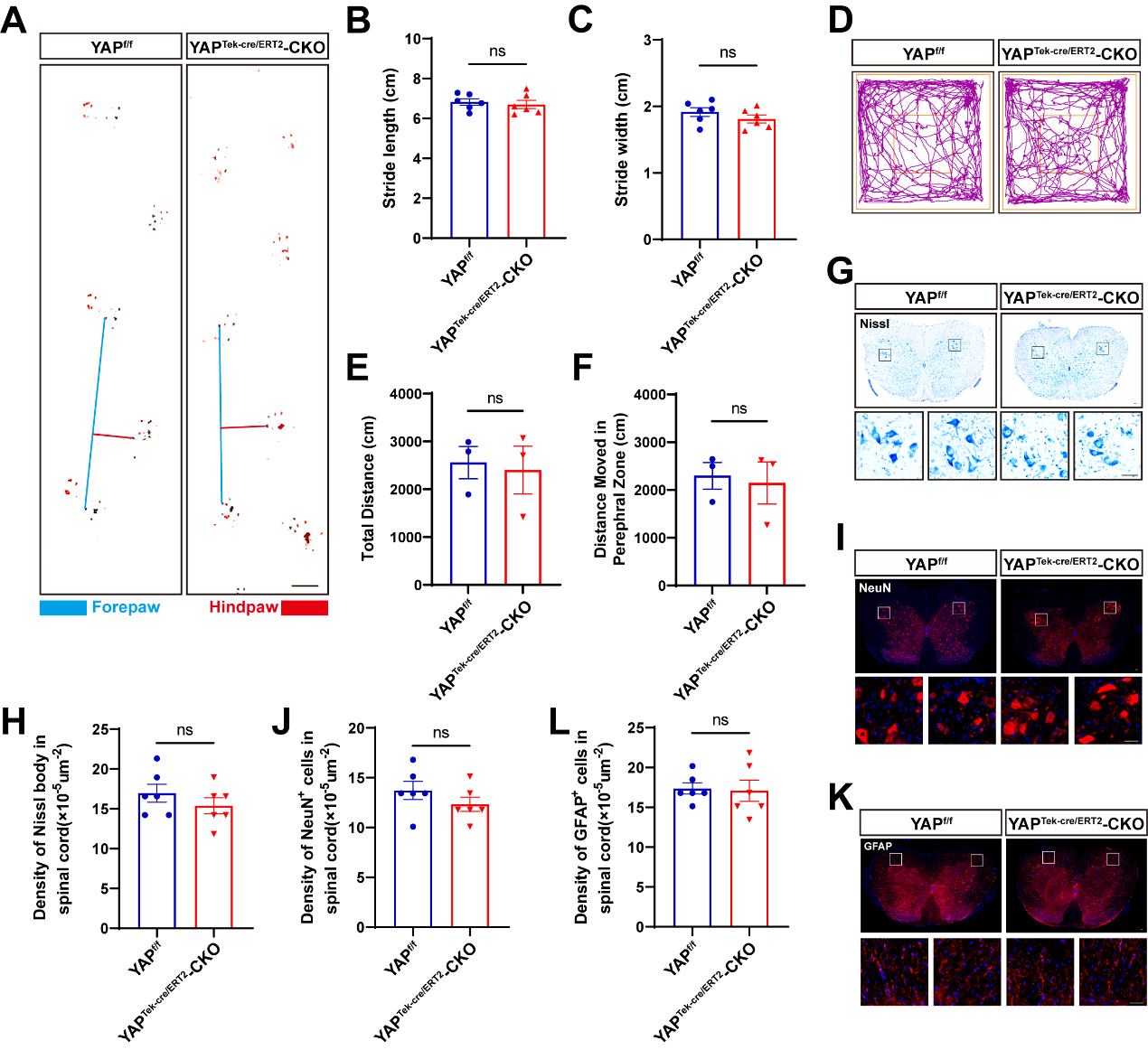
**

**Figure S2. Adult endothelial YAP deficiency didn’t affect the normal motor function of mice.**

**(A)** Representative footprint images of 2 month-YAP^f/f^ and YAP^TEK-cre/ERT2^-CKO mice. Scale bar = 1 cm. **(B, C)** Quantification of stride length (**B**) and stride width (**C**) by footprint analysis in 2 month*-*YAP^f/f^ and YAP^TEK-cre/ERT2^-CKO mice (n = 6 mice per group). **(D)** Representative open field test images of 2 month*-*YAP^f/f^ and YAP^TEK-cre/ERT2^-CKO mice. **(E, F)** Quantification of total distance and distance moved in the peripheral zone by the open field test (n = 3 mice per group). **(G)** Representative Nissl staining images from spinal cord sections of 2 month*-*YAP^f/f^ and YAP^TEK-cre/ERT2^-CKO mice. Scale bar = 50 μm. **(H)** Quantification of Nissl body density as shown in (**G**) (n = 6 sections from 6 mice per group). **(I, K)** Representative immunostaining images of NeuN (**I**) and GFAP (**K**) of spinal cord sections from 2 month*-*YAP^f/f^ and YAP^TEK-cre/ERT2^-CKO mice. **(J, L)** Quantification of NeuN^+^ (**J**) and GFAP^+^ (**L**) cell density as shown in (**I, K**) (n = 6 sections from 6 mice per group). Scale bar = 50 μm. Data were presented as mean ± SEM, two-tailed unpaired Student’s t-test, ^*^ *p* < 0.05, ^**^ *p* < 0.01.

**
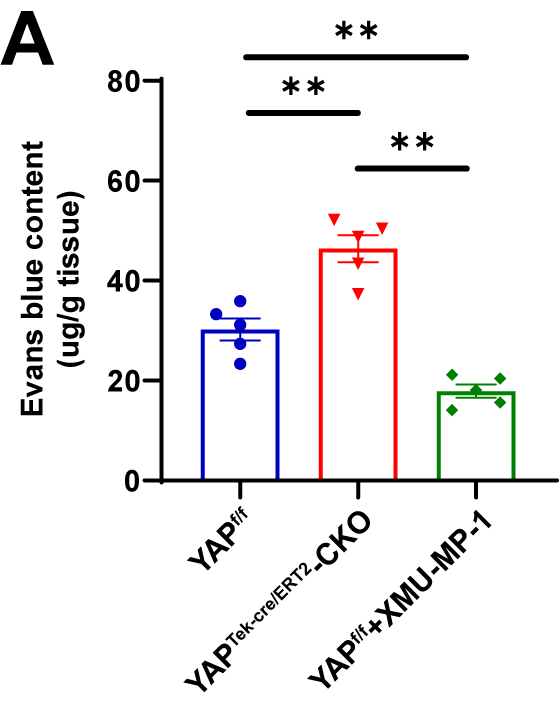
**

**Figure S3. Spectrophotometric quantification of EB extravasation in YAP^f/f^,** **YAP^TEK-cre/ERT2^-CKO and YAP^f/f^ + XMU-MP-1 mice after SCI.**

**(A)** Spectrophotometric quantification of EB extravasation in spinal cord tissue at 7 days after SCI in YAP^f/f^, YAP^TEK-cre/ERT2^-CKO, and YAP^f/f^ + XMU-MP-1 mice. EB concentration was determined by measuring absorbance at 620 nm using a microplate reader and normalized to tissue weight (μg/g tissue) (n = 5 mice per group). Data were presented as mean ± SEM, one-way ANOVA with Tukey’s post hoc test, ^**^ *p* < 0.01.

**
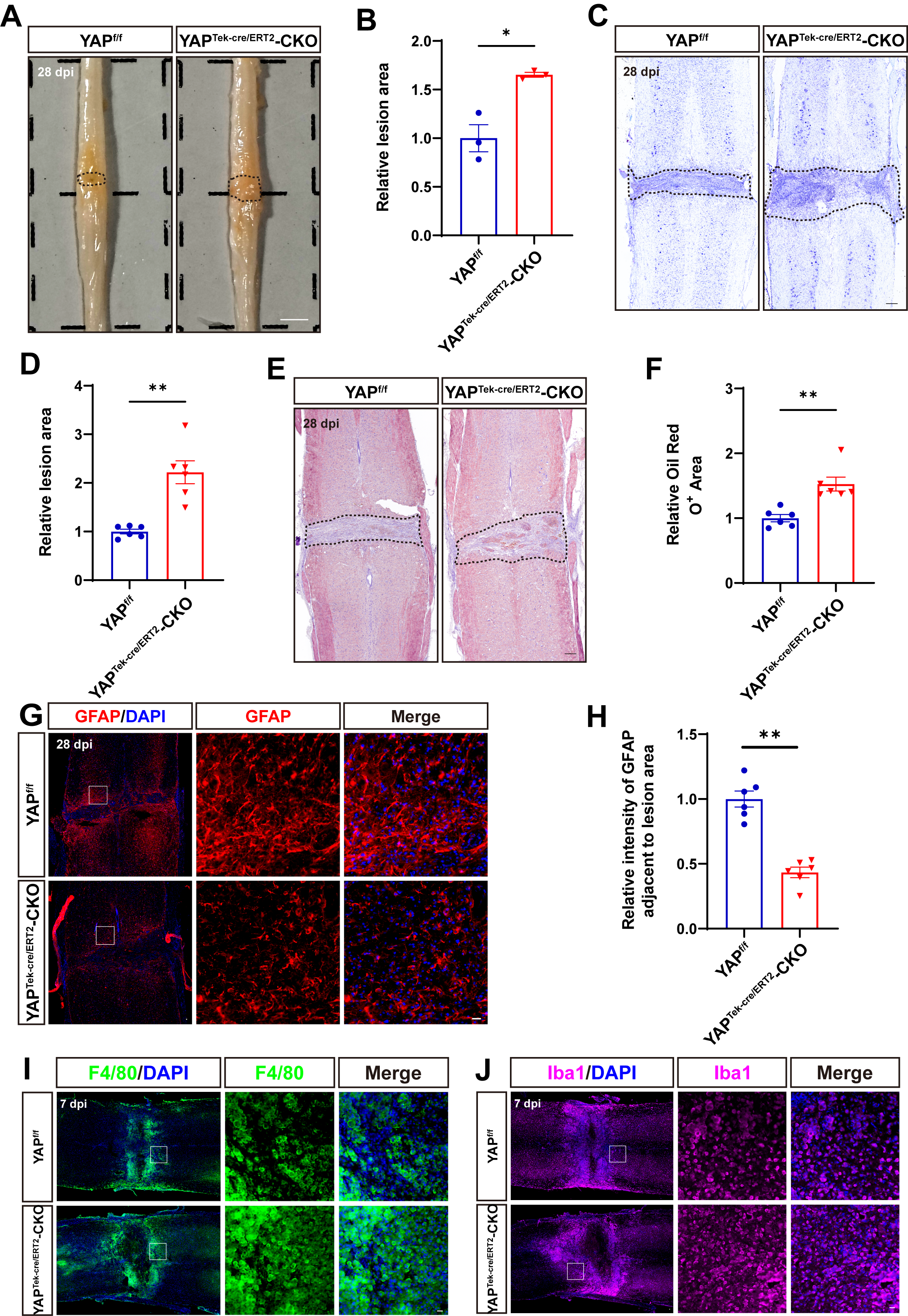
**

**Figure S4. Endothelial YAP deficiency increased lesion area after SCI and impaired the formation of glial scar in mice.**

**(A)** Representative images of the spinal cord, showing lesion size in YAP^f/f^ and YAP^TEK-cre/ERT2^-CKO mice at 28 days after SCI. Scale bar = 200 mm. **(B)** Quantification of lesion size shown in (**A**) (n = 3 mice per group). **(C)** Representative images of Nissl staining showing lesion size in YAP^f/f^ and YAP^TEK-cre/ERT2^-CKO mice at 28 days after SCI. Scale bar = 200 μm. **(D)** Quantification of lesion size from Nissl staining (**C**) (n = 6 sections from 3 mice per group). **(E)** Representative images of Oil Red O staining showing lesion size in YAP^f/f^ and YAP^TEK-cre/ERT2^-CKO mice at 28 days after SCI. Scale bar = 200 μm. **(F)** Quantification of lesion size from Oil Red O staining as shown in (**E**) (n = 6 sections from 3 mice per group). **(G)** Representative images of GFAP immunostaining in YAP^f/f^ and YAP^TEK-cre/ERT2^-CKO mice at 28 days after SCI. Scale bar = 20 μm. **(H)** Quantification of GFAP intensity adjacent to the lesion site as shown in (**G**) (n = 6 sections from 3 mice per group). **(I)** Representative images of F4/80 immunostaining in YAP^f/f^ and YAP^TEK-cre/ERT2^-CKO mice at 7 days after SCI. Scale bar = 20 μm. **(J)** Representative images of Iba1 immunostaining in YAP^f/f^ and YAP^TEK-cre/ERT2^-CKO mice at 7 days after SCI. Scale bar = 20 μm. Data were presented as mean ± SEM, two-tailed unpaired Student’s t-test, ^*^ *p* < 0.05, ^**^ *p* < 0.01.


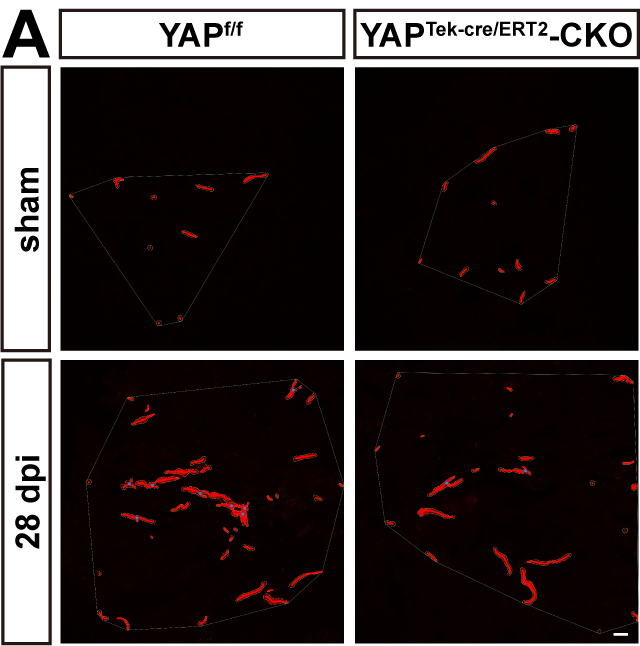


**Figure S5. Representative intermediary images from AngioTool analysis for vessel network quantification.**

**(A)** Representative intermediary images of tdTom^+^ vessels showing vessel segmentation and skeletonization/tracing outputs generated by AngioTool analysis in YAP^f/f^ and YAP^TEK-cre/ERT2^-CKO mice at sham and 28 d after SCI. Scale bar = 20 μm.

**
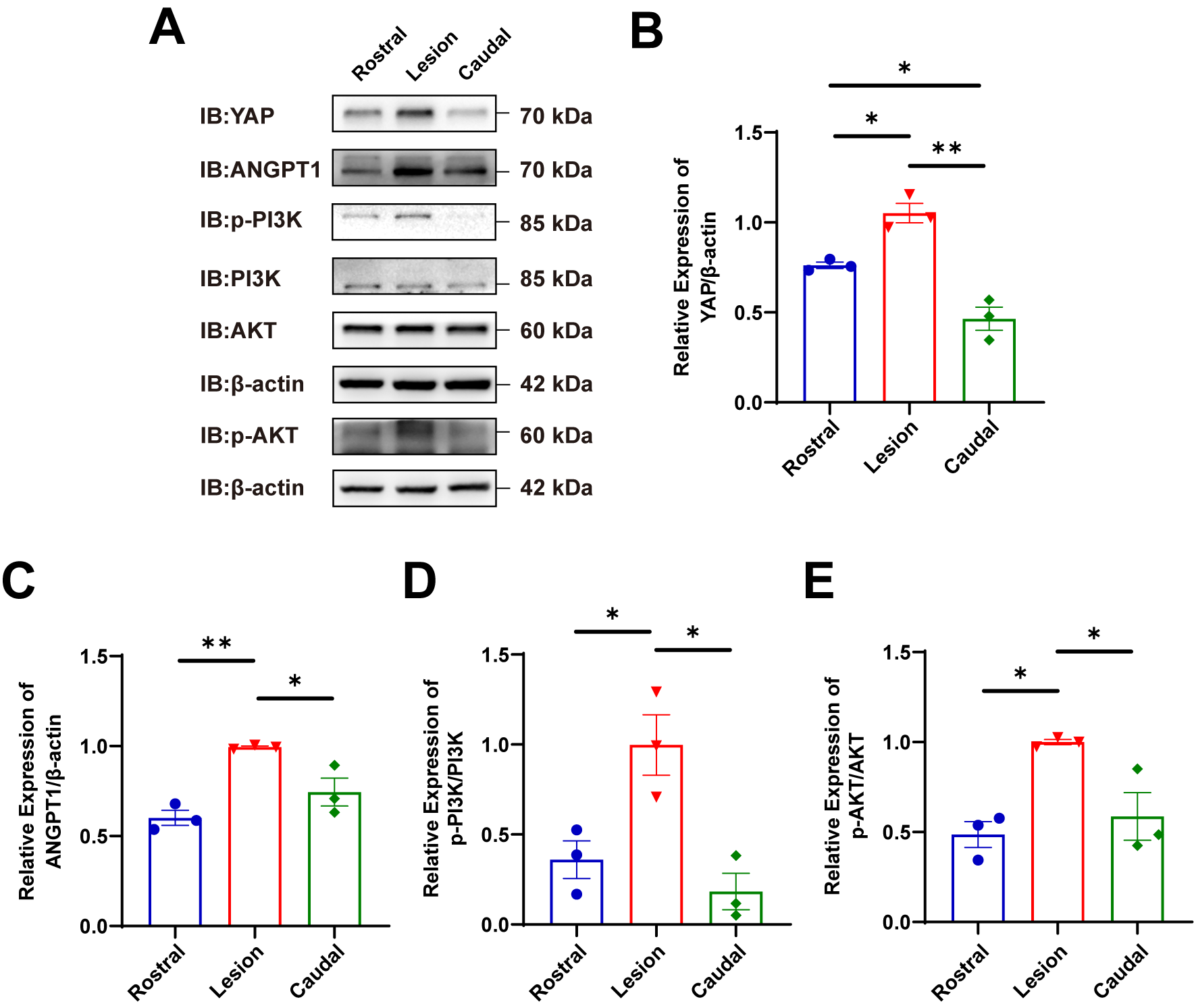
**

**Figure S6.** **Spatially restricted activation of YAP, ANGPT1, and PI3K/AKT signaling at the lesion epicenter in mice after SCI.**

**(A)** WB analysis of the expression of YAP, ANGPT1, p-PI3K, PI3K, p-AKT, and AKT in the rostral segment (+2.5 mm), lesion epicenter, and caudal segment (-2.5 mm) of spinal cords in wide type mice at 7 days after SCI. **(B-E)** Quantitative analysis of the relative expression of YAP (**B**), ANGPT1 (**C**), p-PI3K/PI3K (**D**), and p-AKT/AKT (**E**) as shown in (**A**) (n = 3 blots from 3 mice per group, normalized to lesion epicenter). Data were presented as mean ± SEM, one-way ANOVA with Tukey’s post hoc test, ^*^ *p* < 0.05, ^**^ *p* < 0.01.

**
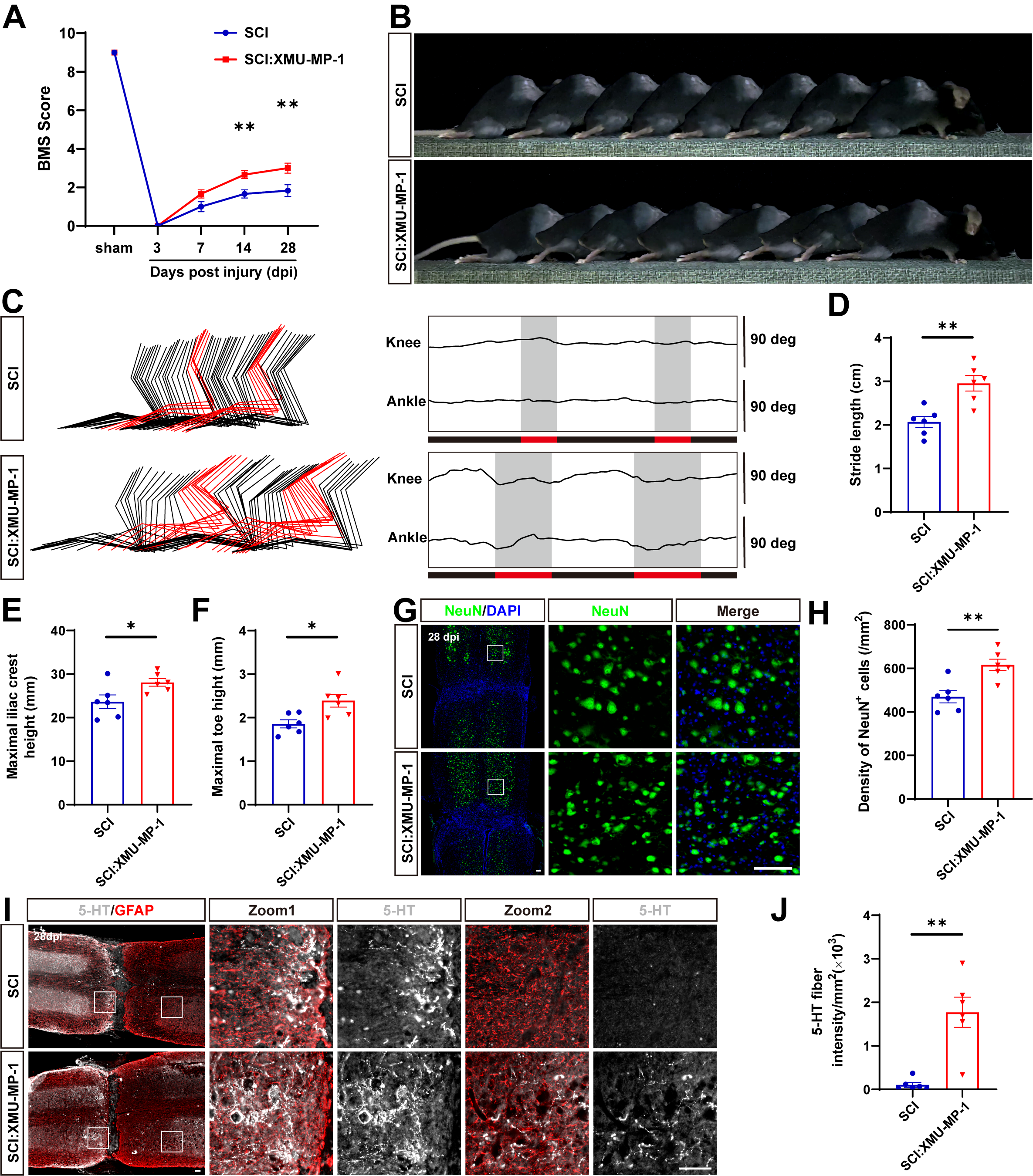
**

**Figure S7. Activation of YAP-associated signaling promoted functional motor recovery and inhibited neurodegeneration in mice after SCI.**

**(A)** BMS score analysis of control and XMU-MP-1-treated mice after SCI (n = 6 mice per group). **(B, C)** Representative chronophotographs of mice, accompanied by corresponding color-coded stick-figure decompositions of hindlimb movements and oscillation traces of knee and ankle joint angles in control and XMU-MP-1-treated mice at 28 days after SCI. **(D-F)** Quantification of stride length (**D**), maximal iliac crest height (**E**), and maximal toe height (**F**) in control and XMU-MP-1-treated mice (n = 6 mice per group). **(G)** Representative images of NeuN (green) immunostaining in control and XMU-MP-1-treated mice at 28 days after SCI. **(H)** Quantitative analysis of the density of NeuN^+^ cells adjacent to the lesion site as shown in (**G**) (n = 6 sections from 6 mice per group). **(I)** Representative images of 5-HT and GFAP immunostaining in control and XMU-MP-1-treated mice at 28 days after SCI. **(J)** Quantitative analysis of 5-HT fiber intensity below the level of SCI as shown in (**I**) (n = 6 sections from 6 mice per group). Scale bar = 100 μm. Data were presented as mean ± SEM, two-way ANOVA with Tukey’s post hoc test (A) and two-tailed unpaired Student’s t-test (D-F, H, and J), ^*^ *p* < 0.05, ^**^ *p* < 0.01.

**
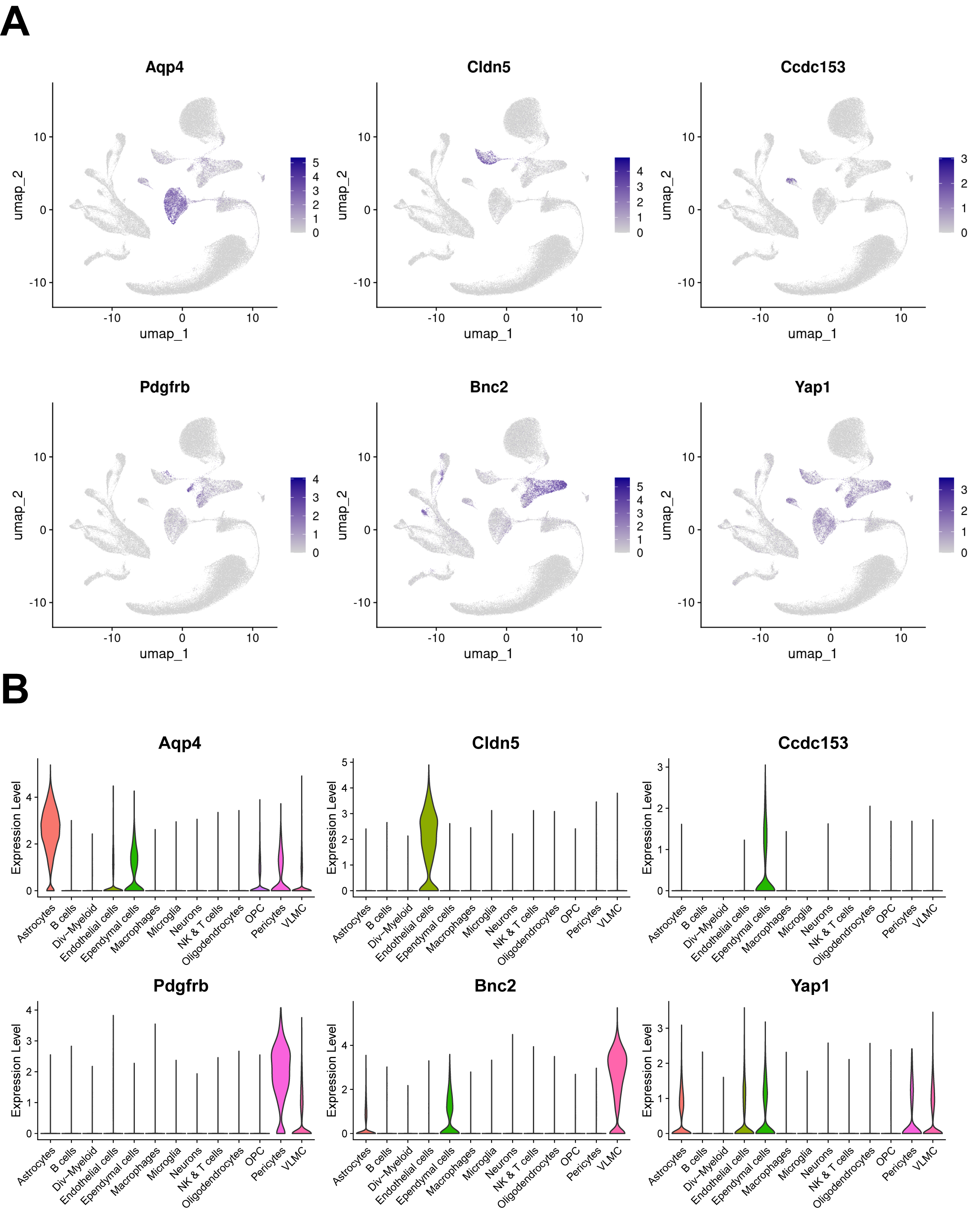
**

**Figure S8.** **Marker gene analysis identified the expression pattern of Yap1 in cluster cell types in mice after SCI.**

**(A)** UMAP feature plots showing the normalized expression of representative marker genes (*Aqp4, Cldn5, Ccdc153, Pdgfrb,* and *Bnc2*) and *Yap1* across different cell populations after SCI, based on the publicly available scRNA-seq dataset (GSE234774). **(B)** Violin plots showing the expression distribution of representative marker genes and *Yap1* across annotated cell types.

**
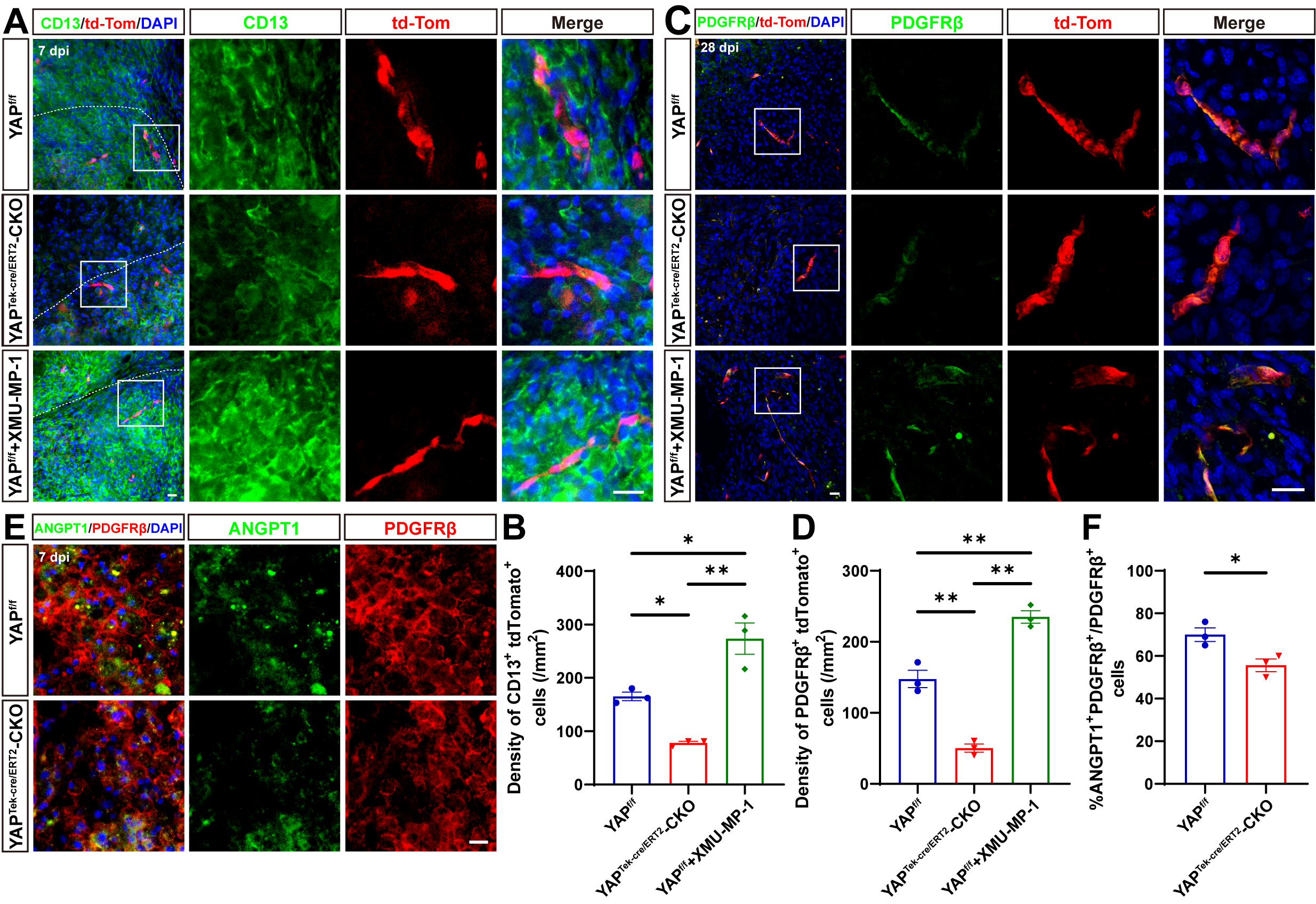
**

**Figure S9.** **Activation of YAP-associated signaling by XMU-MP-1 promoted pericyte coverage of spinal microvessels in mice after SCI.**

**(A)** Representative immunofluorescence staining images of CD13 (green) and tdTom^+^ cells in YAP^f/f^-tdTom, YAP^TEK-cre/ERT2^-CKO-tdTom, and YAP^f/f^-tdTom + XMU-MP-1 mice at 7 days after SCI. **(B)** Quantitative analysis of the density of CD13^+^ in tdTom^+^ cells as shown in (**A**) (n = 3 sections from 3 mice per group). **(C)** Representative immunofluorescence staining images of PDGFRβ (green) and tdTom^+^ cells in YAP^f/f^-tdTom, YAP^TEK-cre/ERT2^-CKO-tdTom, and YAP^f/f^-tdTom + XMU-MP-1 mice at 28 days after SCI. **(D)** Quantitative analysis of the density of PDGFRβ^+^ in tdTom^+^ cells as shown in (**C**) (n = 3 sections from 3 mice per group). **(E)** Representative immunofluorescence staining images of ANGPT1 (green) and PDGFRβ cells in YAP^f/f^ and YAP^TEK-cre/ERT2^-CKO mice at 7 days after SCI. **(F)** Quantitative analysis of ANGPT1^+^PDGFRβ^+^ cells as a percentage of PDGFRβ^+^ cells as shown in (**E**) (n = 3 sections from 3 mice per group). Scale bar = 20 μm. Data were presented as mean ± SEM, one-way ANOVA with Tukey’s post hoc test (B and D) and two-tailed unpaired Student’s t-test (F), ^*^ *p* < 0.05, ^**^ *p* < 0.01.

Table S1. Primary and secondary antibodies used in this study.

| **Antibody** | **Host** | **Application** | **Dilution** | **Supplier** | **Catalog No.** |
| --- | --- | --- | --- | --- | --- |
| YAP | Rabbit | WB | 1:1000 | Cell Signaling Technology (CST) | #14074 |
| YAP | Rabbit | WB  (Fig. 7D) | 1:1000 | GeneTex | GTX129151 |
| β-Tubulin | Rabbit | WB | 1:10000 | HUABIO | ET1602-4 |
| ZO-1 | Rabbit | WB | 1:1000 | Invitrogen | 61-7300 |
| Occludin | Rabbit | WB | 1:1000 | Proteintech | 27260-1-AP |
| HIF-1α | Rabbit | WB | 1:1000 | Proteintech | 20960-1-AP |
| ANGPT1 | Rabbit | WB | 1:1000 | Proteintech | 27093-1-AP |
| p-PI3K | Rabbit | WB | 1:1000 | CST | #4228 |
| PI3K | Rabbit | WB | 1:1000 | CST | #4257 |
| p-AKT | Rabbit | WB | 1:1000 | CST | #13038 |
| AKT | Mouse | WB | 1:1000 | CST | #2920 |
| GAPDH | Rabbit | WB | 1:5000 | HUABIO | ET1601-4 |
| AQP4 | Rabbit | WB | 1:1000 | Proteintech | 16473-1-AP |
| β-Actin | Mouse | WB | 1:10000 | HUABIO | EM21002 |
| YAP | Rabbit | IF | 1:400 | CST | #14074 |
| ZO-1 | Rabbit | IF | 1:400 | Invitrogen | 61-7300 |
| F4/80 | Rabbit | IF | 1:400 | Proteintech | 28463-1-AP |
| Iba1 | Rabbit | IF | 1:400 | Abcam | ab178846 |
| NF | Rabbit | IF | 1:300 | Abcam | ab8135 |
| Ki67 | Rabbit | IF | 1:400 | CST | #9129S |
| AQP4 | Rabbit | IF | 1:400 | Proteintech | 16473-1-AP |
| ANGPT1 | Rabbit | IF | 1:400 | Proteintech | 27093-1-AP |
| 5-HT | Rabbit | IF | 1:5000 | Immunostar | 20080 |
| CD13 | Rabbit | IF | 1:100 | Abmart | TB5828 |
| NeuN | Mouse | IF | 1:400 | CST | #94403 |
| GFAP | Mouse | IF | 1:400 | Millipore | MAB360 |
| PDGFRβ | Mouse | IF | 1:400 | Abmart | M006509 |
| CD31 | Goat | IF | 1:200 | R&D Systems | AF3628 |
| Occludin | Goat | IF | 1:400 | Abmart | PGG039 |
| Goat anti-rabbit IgG-HRP | Goat | WB secondary | 1:10000 | Pierce | #31420 |
| Goat anti-mouse IgG-HRP | Goat | WB secondary | 1:10000 | Pierce | #31460 |
| Goat anti-rabbit IgG Alexa Fluor 488 | Goat | IF  secondary | 1:1000 | Invitrogen | A32731 |
| Goat anti-rabbit IgG Alexa Fluor 568 | Goat | IF  secondary | 1:1000 | Invitrogen | A11011 |
| Goat anti-rabbit IgG Alexa Fluor 647 | Goat | IF  secondary | 1:1000 | Invitrogen | A21245 |
| Goat anti-mouse IgG Alexa Fluor 546 | Goat | IF  secondary | 1:1000 | Invitrogen | A11030 |
| Donkey anti-goat IgG Alexa Fluor 633 | Donkey | IF  secondary | 1:1000 | Invitrogen | A21082 |
| Donkey anti-rabbit IgG Alexa Fluor 546 | Donkey | IF  secondary | 1:1000 | Invitrogen | A10040 |
| Goat anti-rabbit IgG Alexa Fluor 647 | Goat | IF  secondary | 1:1000 | Beyotime | A0468 |
| Goat anti-mouse IgG Alexa Fluor 647 | Goat | IF  secondary | 1:1000 | Beyotime | A0473 |
| Goat anti-mouse IgG Alexa Fluor 488 | Goat | IF  secondary | 1:1000 | Beyotime | A0428 |
